# Integrative analysis of GWAS, Bayesian fine-mapping, Mendelian randomization and colocalization reveals genetic determinants underlying milk-related traits in dairy cattle

**DOI:** 10.1186/s12711-025-01028-3

**Published:** 2026-01-03

**Authors:** Jun Teng, Xiuxin Zhao, Qingxia Yan, Jian Yang, Fen Pei, Xinyi Zhang, Chongwei Duan, Zhujun Chen, Qianwen Xu, Yan Liu, Guanghui Xue, Shuwen Xia, Huili Wang, Yao Gu, Lingzhao Fang, Huiming Liu, Hongding Gao, Jing An, Li Jiang, Chao Ning, Rongling Li, Yundong Gao, Xiao Wang, Jianbin Li, Qin Zhang

**Affiliations:** 1 Shandong Provincial Key Laboratory for Livestock Germplasm Innovation and Utilization, College of Animal Science and Technology, Shandong Agricultural University, Tai’an, 271018 Shandong China; 2 Institute of Animal Science and Veterinary Medicine, Shandong Academy of Agricultural Sciences, Jinan, 250100 China; 3 Dairy Association of China, Beijing, 100193 China; 4 Shandong OX Livestock Breeding Co., Ltd., Jinan, 250100 China; 5 Institute of Animal Science, Jiangsu Academy of Agricultural Sciences, Nanjing, 210014 China; 6 Modern Farming (Group) Co., Ltd., Ma’anshan, 243000 China; 7 Center for Quantitative Genetics and Genomics, Aarhus University, 8000 Aarhus, Denmark; 8 Natural Resources Institute Finland (Luke), 31600 Jokioinen, Finland; 9 CNRS, INRAE, Université Evry, Institute of Plant Sciences Paris-Saclay (IPS2), Université Paris-Saclay, 91405 Orsay, France; 10 College of Animal Science and Technology, China Agricultural University, Beijing, 100193 China

## Abstract

**Background:**

Genome-wide association studies (GWAS) has identified many genetic variants associated with milk-related traits in dairy cattle. However, the causal variants or genes remain largely unknown. In this study, using a large population (> 10,000 individuals) of Chinese Holstein cattle, we performed GWAS for six milk-related traits (milk yield, fat percentage, protein percentage, fat yield, protein yield, and somatic cell score) and subsequently prioritized putative causal variants by multi-trait Bayesian fine-mapping and examined the causal genes by Mendelian randomization (MR) analysis incorporating GWAS and *cis*-eQTL summary data from CattleGTEx. We also conducted a colocalization analysis to identify the share putative causal variants behind the milk-related traits and gene expressions.

**Results:**

A total of 9,688 genome-wide significant SNPs (*P* < 1.2 × 10^−7^) were identified across the GWAS results for six milk-related traits, and these SNPs were distributed in 25 unique QTL regions. Subsequently, the multi-trait Bayesian fine-mapping identified 211 independent credible sets (CS) containing putative causal variants within these QTL regions. Among these CSs, 189 CSs were significantly associated with at least one trait (average *lfsr* < 0.01). Notably, the lead SNPs within these significant CSs included 3 missense variants and 62 non-coding transcript variants. The MR analysis detected 268 causal associations between gene expression and milk-related traits. The colocalization analysis identified two regions containing common putative causal variants for one or multiple milk-related traits and the expressions of some genes.

**Conclusions:**

Our integrative analysis of GWAS, Bayesian fine-mapping, MR, and colocalization further confirmed the well-known causal associations of *DGAT1* and *GHR* and the milk-related traits. In addition, we revealed some novel potential causal genes, including *AHNAK*, *ARHGEF2*, *SOX13*, *FDPS*, *SCGB2A2*, and *MROH2B*. These results enhance our understanding of genetic mechanisms underlying the milk-related traits in dairy cattle.

**Supplementary Information:**

The online version contains supplementary material available at 10.1186/s12711-025-01028-3.

## Background

Genome-wide association studies (GWAS) have identified hundreds of thousands of genetic variants associated with milk-related traits across different dairy cattle populations [1–4]. However, the associated variants from GWAS are largely in linkage disequilibrium (LD) with the causal variants, which severely hinder the efficient identification of causal variants. In addition, insufficient sample sizes and marker densities in most GWAS limited the power and resolution of detecting causal variants. With rapid reduction of sequencing cost and the development of the 1000 Bull Genomes project [5], an increasing number of studies have leveraged imputed sequence-level data for GWAS in large populations, which greatly improved the power and resolution of uncovering associated variants. However, identifying truly causal variants among the large number of associated loci remains a major challenge. One strategy often used for identifying casual variants is to selecting the top SNP with the strongest GWAS association evidence in a QTL region as a candidate causal variant. However, owing to high LD between multiple significant SNPs within a QTL region, the top SNP in a QTL region is often non-causal variant [6, 7]. The recently developed Bayesian fine-mapping methods, such as BIMBAM [8], FINEMAP [9], and SuSiE [10], address this issue by estimating posterior inclusion probability (PIP) of each SNP within a Bayesian framework while accounting for LD [11], thereby improving causal inference. While having been successfully applied to identify numerous putative causal variants influencing human diseases [12–14], Bayesian fine-mapping methods have also shown promise in cattle. For instance, Jiang et al. [15] developed a Bayesian Fine-MAPping approach (BFMAP) and fine-mapped 308 association signals for 32 agronomic traits in Holstein cattle. Gualdrón et al. [16] used SuSiE to identify 616 candidate causal variants for 11 linear classification traits in Belgian Blue beef cattle.

Although Bayesian fine-mapping methods can help us to identify putative causal variants after GWAS, linking the effect of these variants to gene function is not straightforward without additional data, especially, as the majority of putative causal variants fall into non-coding or intergenic regions. Therefore, integrating additional data such as expression quantitative trait loci (eQTL) is essential to connect causal variant effects to gene function [17]. To leverage GWAS and eQTLs data to reveal genetic determinants, transcriptome-wide association studies (TWAS) have been proposed to identify gene-trait associations [18]. However, while TWAS can identify genes whose expression is significantly associated with complex traits, they are not designed to quantify the magnitude of the causal effect and unable to distinguish causation from horizontal pleiotropy [19]. In contrast, Mendelian randomization (MR) combines GWAS and eQTL data to determine the causal relationships between an exposure variable (e.g., gene expression) and an outcome variable (e.g., complex trait), using SNPs as instrumental variables (IV). Additionally, numerous GWAS and eQTL studies [20–22] have demonstrated that many GWAS variants are also eQTLs, i.e., a variant can be commonly associated with a trait and the expression of a gene. Colocalization analysis has been proposed to assess the causality of such shared associations, i.e., whether this variant is a shared causal variant for the trait and the gene expression [23]. Some of the above-mentioned methods have been used to reveal the genetic determinants of complex traits in cattle. Forutan et al. [24] used the SMR method with single-trait GWAS summary data and *cis*-eQTL data from blood and identified 8 genes with causal associations to cattle fertility traits. Similarly, Cai et al. [25] applied colocalization analysis and found that eQTLs for 176 genes (eGenes, i.e., genes regulated by eQTLs) across three tissues (longissimus dorsi muscle, backfat, and liver) colocalized with variants associated with growth and carcass traits in Simmental beef cattle.

**Fig. 1:**
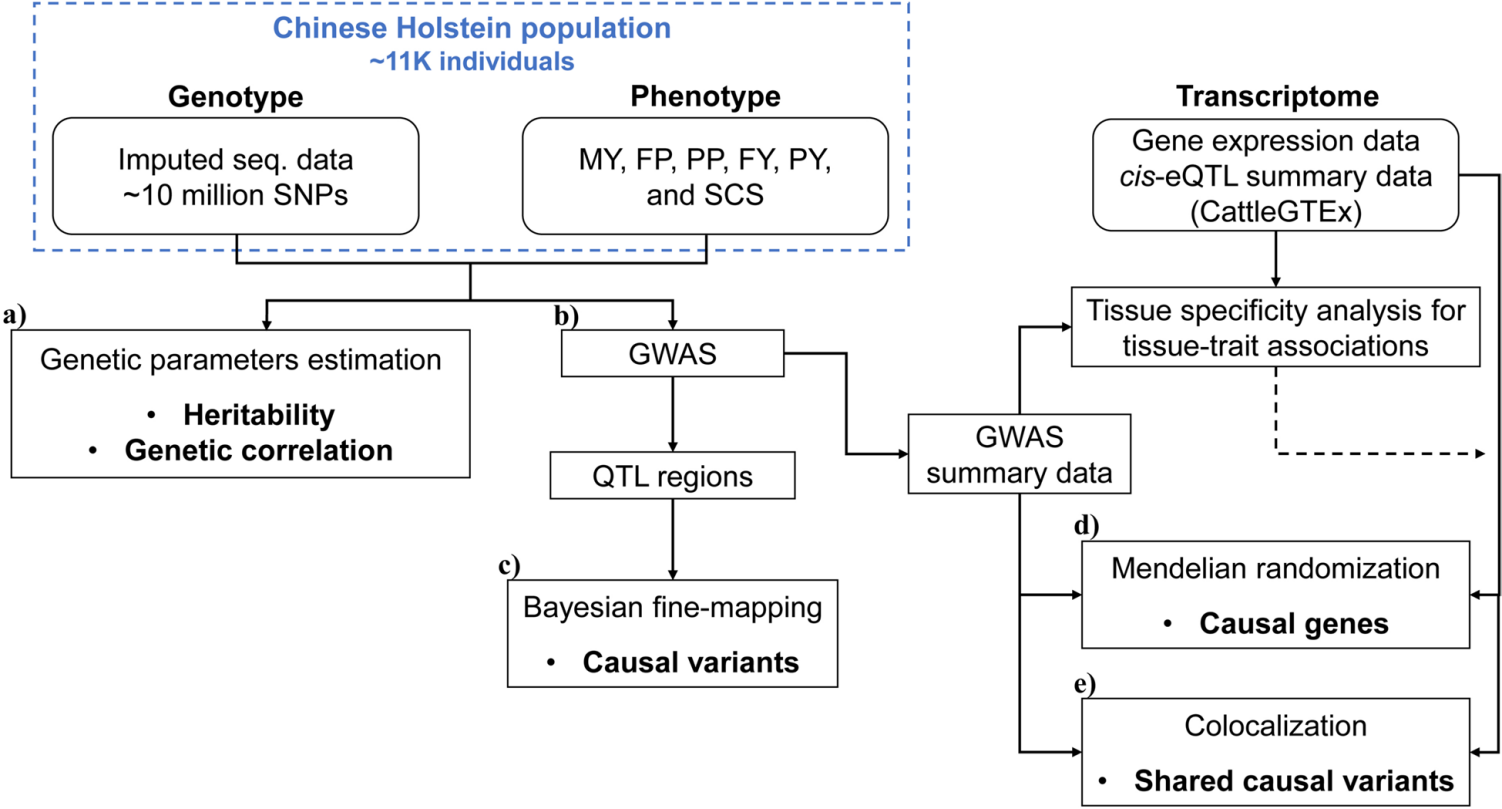
Schematic diagram of the study design. (a) SNP-based heritability and genetic correlation estimation. (b) GWAS was performed to identify QTL regions and provide summary statistic data for the subsequent analysis. (c) Bayesian fine-mapping was performed to identify putative causal variants within QTL regions. (d) Mendelian randomization was performed to identify causal genes throughout the whole genome using the GWAS summary data and *cis*-eQTL summary data from CattleGTEx. Tissue specificity analysis (using MAGMA) was conducted for selecting tissues in which the tissue specific gene sets are most relevant to the traits involved in this study. (e) Colocalization was performed to identify the share causal variants between the traits and gene expressions. Again, only tissues most relevant to the traits are considered. MY, milk yield; FP, fat percentage; PP, protein percentage; FY, fat yield; PY, protein yield; SCS, somatic cell score

Nevertheless, few studies have interactively applied these methods (e.g., Bayesian fine-mapping, MR and colocalization) for precise identification of causal variants or genes. In this study, we performed an integrative analysis of GWAS, Bayesian fine-mapping, MR, and colocalization for six milk-related traits of dairy cattle in a large population (> 10,000 individuals) of Chinese Holsteins (Fig. 1 for a schematic illustration of the study design). We first performed imputed sequence-based GWAS to identify QTL regions, followed by Bayesian fine-mapping to pinpoint the putative causal variants within these regions. To explore gene-level regulatory mechanisms, we leveraged the eQTL data from the CattleGTEx resource [21] to conduct MR to identify the causal relationship between gene expressions and the milk-related traits. Finally, we performed colocalization analysis to determine the shared causal variants for both gene expressions and traits. Our results comprehensively revealed the QTLs and putative causal variants or genes underlying the studied traits, thereby enhancing our understanding of genetic mechanisms of milk-related traits in dairy cattle.

## Materials and methods

### Study population, phenotype, and genotype data

A population of 11,059 individuals of Chinese Holstein cows, born between 2003 and 2021 from 158 farms, was used to investigate genetic architecture and infer putative causal variants for six milk-related traits, i.e., milk yield (MY), fat percentage (FP), protein percentage (PP), fat yield (FY), protein yield (PY), and somatic cell score (SCS). The estimated breeding values (EBVs) for each individual were provided by the Chinese Dairy Association, which were based on a multi-trait and multi-lactation test-day random regression model. We converted these EBVs to de-regressed proofs (DRP) using the method of VanRaden et al. [26, 27] and considered DRPs as the pseudo phenotypes for further analysis. The summary statistics of all traits were shown in Additional file 2 Table S1. Genotyping was performed using three SNP arrays with varying densities: Illumina Bovine SNP50 BeadChip (50 K), GGP Bovine 100K (100 K), and GGP Bovine HDv3 (150 K). Specifically, 5028 cows were genotyped with the 50 K chip, 2373 cows with the 100 K chip, and 3658 cows with the 150 K chip. The genomic positions of SNPs of the three chips were unified using the bovine reference genome (ARS-UCD1.2). We constructed a reference panel based on sequence data of 3530 cattle (average sequencing depth > 10 ×) obtained from the NCBI Sequence Read Archive (SRA) database (https://www.ncbi.nlm.nih.gov/sra). We removed non biallelic SNPs and SNPs located on the sex chromosomes in the reference panel, resulting in a total of 28,166,177 SNPs for imputation. Then, we used Beagle v5.1 [28] to directly impute the chip level genotypes to whole-genome sequence level. The imputation accuracies for the three chips were assessed in our previous study [29], where the imputation for the same three chips was conducted using the same reference panel. After imputation, SNPs with minor allele frequency (MAF) < 0.05 or with *P*-value of Hardy–Weinberg Equilibrium test < 1 × 10^−6^ were excluded, resulting in 10,315,288 SNPs for subsequent analysis.

### SNP-based heritability and genetic correlation estimation

The following univariate linear mixed model was used to estimate the heritability for each trait:

1 $$ \begin{gathered} {\mathbf{y}} = {\mathbf{\mu }} + {\mathbf{Za}} + {\mathbf{e}}, \hfill \\ {\mathbf{a}}\sim N\left( {\mathbf{0},{\mathbf{G}}\sigma _{{\text{a}}}^{2} } \right);{\mathbf{e}}\sim N\left( {\mathbf{0},{\mathbf{I}}\sigma _{{\text{e}}}^{2} } \right) \hfill \\ \end{gathered} $$

where $\mathbf{y}$ is the vector of phenotypic values (DRP) of the trait, ${\varvec{\upmu}}$ is the overall means, $\mathbf{a}$ is the vector of additive genetic effects, $\mathbf{Z}$ is the design matrix associating **a** with $\mathbf{y}$; ${\sigma }_{\text{a}}^{2}$ is the additive genetic variance, $\mathbf{G}=\frac{\mathbf{W}{\mathbf{W}}^{\mathbf{^{\prime}}}}{\sum 2{p}_{j}\left(1-{p}_{j}\right)}$ is the genomic relationship matrix (GRM) defined by VanRaden’s method 1 [30] constructed using 10,315,288 SNPs from imputed sequence data after quality control, $\mathbf{W}$ being the centralized SNP genotype matrix with element *w*_*ij*_ = *m*_*ij*_—2*p*_*j*_, *m*_*ij*_ being the genotype code (0, 1, and 2 for genotypes AA, AB, and BB, respectively) for individual *i* and SNP *j*, ${p}_{j}$ being the MAF of SNP *j*; $\mathbf{e}$ is the vector of the random residuals, $\mathbf{I}$ is an identity matrix, and ${\sigma }_{\text{e}}^{2}$ is the residual variance. The heritability estimate of the trait was calculated as ${h}^{2}=\frac{{\sigma }_{\text{a}}^{2}}{{\sigma }_{\text{p}}^{2}}$, ${\sigma }_{\text{p}}^{2}={\sigma }_{\text{a}}^{2}+{\sigma }_{\text{e}}^{2}$ is the phenotypic variance. The standard error (SE) for the estimated heritability was calculated as $SE\left({h}^{2}\right)=\sqrt{{({h}^{2})}^{2}\left[\frac{Var({\sigma }_{\text{a}}^{2})}{{({\sigma }_{\text{a}}^{2})}^{2}}+\frac{Var({\sigma }_{\text{p}}^{2})}{{({\sigma }_{\text{p}}^{2})}^{2}}-2\frac{Cov({\sigma }_{\text{a}}^{2},{\sigma }_{\text{p}}^{2})}{{\sigma }_{\text{a}}^{2}{\sigma }_{\text{p}}^{2}}\right]}$, where $Var({\sigma }_{\text{a}}^{2})$ and $Var({\sigma }_{\text{p}}^{2})$ are sampling variances of ${\sigma }_{\text{a}}^{2}$ and ${\sigma }_{\text{p}}^{2}$, $Cov({\widehat{\sigma }}_{\text{a}}^{2},{\widehat{\sigma }}_{\text{p}}^{2})$ is covariance between ${\sigma }_{\text{a}}^{2}$ and ${\sigma }_{\text{p}}^{2}$.

The following bivariate linear mixed model was used to estimate genetic correlation between two traits:

2 $$ \begin{gathered} \left[ {\begin{array}{*{20}c} {{\mathbf{y}}_{1} } \\ {{\mathbf{y}}_{2} } \\ \end{array} } \right] = \left[ {\begin{array}{*{20}c} {{\mathbf{\mu }}_{1} } \\ {{\mathbf{\mu }}_{2} } \\ \end{array} } \right] + \left[ {\begin{array}{*{20}c} {{\mathbf{Z}}_{1} } & 0 \\ 0 & {{\mathbf{Z}}_{2} } \\ \end{array} } \right]\left[ {\begin{array}{*{20}c} {{\mathbf{a}}_{1} } \\ {{\mathbf{a}}_{2} } \\ \end{array} } \right] + \left[ {\begin{array}{*{20}c} {{\mathbf{e}}_{1} } \\ {{\mathbf{e}}_{2} } \\ \end{array} } \right] \hfill \\ \left[ {\begin{array}{*{20}c} {{\mathbf{a}}_{1} } \\ {{\mathbf{a}}_{2} } \\ \end{array} } \right]\sim \varvec{N}\left( {\mathbf{0},{\mathbf{M}} \otimes {\mathbf{G}}} \right),{}\left[ {\begin{array}{*{20}c} {{\mathbf{e}}_{1} } \\ {{\mathbf{e}}_{2} } \\ \end{array} } \right]\sim \varvec{N}\left( {0,{\mathbf{R}} \otimes {\mathbf{I}}} \right) \hfill \\ \end{gathered} $$

where all terms are the same as those in Model (1) with the subscripts 1 and 2 refer to trait 1 and trait 2, respectively, $$\mathbf{M}=\left[\begin{array}{cc}{\sigma }_{{\text{a}}_{1}}^{2}& {\sigma }_{{\text{a}}_{12}}\\ {\sigma }_{{\text{a}}_{12}}& {\sigma }_{{\text{a}}_{2}}^{2}\end{array}\right]$$ with ${\sigma }_{{\text{a}}_{1}}^{2}$ and ${\sigma }_{{\text{a}}_{2}}^{2}$ being the additive genetic covariance and ${\sigma }_{{\text{a}}_{12}}$ being the additive genetic variance–covariance for the two traits, and $$\mathbf{R}=\left[\begin{array}{cc}{\sigma }_{{\text{e}}_{1}}^{2}& {\sigma }_{{\text{e}}_{12}}\\ {\sigma }_{{\text{e}}_{12}}& {\sigma }_{{\text{e}}_{2}}^{2}\end{array}\right]$$ with ${\sigma }_{{\text{e}}_{1}}^{2}$ and ${\sigma }_{{\text{e}}_{2}}^{2}$ being the residual variances and ${\sigma }_{{\text{e}}_{12}}$ being the residual covariance for the two traits. The genetic correlation was calculated as ${r}_{g}=\frac{{\sigma }_{{\text{a}}_{12}}}{\sqrt{{\sigma }_{{\text{a}}_{1}}^{2}{\sigma }_{{\text{a}}_{2}}^{2}}}$. The SE for the estimated genetic correlation was calculated as

$$ \begin{gathered} SE\left( {r_{g} } \right) = \hfill \\ \sqrt {\left( {r_{g} } \right)^{2} \begin{array}{*{20}l} {\left[ {\frac{{Var\left( {\sigma _{{{\text{a}}_{{12}} }} } \right)}}{{\left( {\sigma _{{{\text{a}}_{{12}} }} } \right)^{2} }} + \frac{{Var\left( {\sigma _{{{\text{a}}_{1} }}^{2} } \right)}}{{4\left( {\sigma _{{{\text{a}}_{1} }}^{2} } \right)^{2} }} + \frac{{Var\left( {\sigma _{{{\text{a}}_{2} }}^{2} } \right)}}{{4\left( {\sigma _{{{\text{a}}_{2} }}^{2} } \right)^{2} }}} \right.} \hfill \\ { - \frac{{Cov\left( {\sigma _{{{\text{a}}_{{12}} }} ,\sigma _{{{\text{a}}_{1} }}^{2} } \right)}}{{\sigma _{{{\text{a}}_{{12}} }} \sigma _{{{\text{a}}_{1} }}^{2} }} - \frac{{Cov\left( {\sigma _{{{\text{a}}_{{12}} }} ,\sigma _{{{\text{a}}_{2} }}^{2} } \right)}}{{\sigma _{{{\text{a}}_{{12}} }} \sigma _{{{\text{a}}_{2} }}^{2} }}} \hfill \\ {\left. { + \frac{{Cov\left( {\sigma _{{{\text{a}}_{1} }}^{2} ,\sigma _{{{\text{a}}_{2} }}^{2} } \right)}}{{2\sigma _{{{\text{a}}_{1} }}^{2} \sigma _{{{\text{a}}_{2} }}^{2} }}} \right]} \hfill \\ \end{array} } \hfill \\ \end{gathered} $$

$Var({\sigma }_{{\text{a}}_{1}}^{2})$, $Var({\sigma }_{{\text{a}}_{2}}^{2})$, and $Var({\sigma }_{{\text{a}}_{12}})$ are sampling variances of ${\sigma }_{{\text{a}}_{1}}^{2}$, ${\sigma }_{{\text{a}}_{2}}^{2}$, and ${\sigma }_{{\text{a}}_{12}}$, $Cov\left({\sigma }_{{\text{a}}_{1}}^{2},{\sigma }_{{\text{a}}_{2}}^{2}\right)$, $Cov\left({\sigma }_{{\text{a}}_{12}},{\sigma }_{{\text{a}}_{1}}^{2}\right)$, and $Cov\left({\sigma }_{{\text{a}}_{12}},{\sigma }_{{\text{a}}_{2}}^{2}\right)$ are covariances between ${\sigma }_{{\text{a}}_{1}}^{2}$, ${\sigma }_{{\text{a}}_{2}}^{2}$, and ${\sigma }_{{\text{a}}_{12}}$.

The variance and covariance components involved in these two models were estimated by residual maximum likelihood algorithm using GCTA v1.94.1 [31].

### Genome-wide association studies

GWAS was performed using the GMAT software (https://github.com/chaoning/GMAT) based on the linear mixed model,

3 $$ \begin{gathered} {\mathbf{y}} = {\mathbf{\mu }} + {\mathbf{x}}\beta + {\mathbf{Za}} + {\mathbf{e}}, \hfill \\ {\mathbf{a}}\sim N\left( {0,{\mathbf{G}}\sigma _{{\text{a}}}^{2} } \right);{\mathbf{e}}\sim N\left( {0,{\mathbf{I}}\sigma _{{\text{e}}}^{2} } \right) \hfill \\ \end{gathered} $$

where $\mathbf{x}$ is the vector of genotypes of a candidate SNP (coded as 0, 1 or 2) and $\beta$ is the regression coefficient; the rest terms ($\mathbf{y}$, ${\varvec{\upmu}}$, $\mathbf{Z}$, $\mathbf{a}$, etc.) are the same as those in Eq. (1). We employed Bonferroni correction to control false-positive rates from multiple testing. The genome-wide significance threshold was set at 0.05/N, where N represents the number of effective SNPs. The effective SNPs were defined as those that were not in high LD (*r*^2^ < 0.2) with each other. To obtain these SNPs, we performed LD pruning using PLINK v1.90 [32] using command “–indep-pairwise 50 5 0.2” [33, 34]. After LD pruning, 414,447 SNPs were remained, and thus the genome-wide significance threshold was *P* < 1.2 × 10^−7^ (= 0.05/414,447).

### Multi-trait Bayesian fine-mapping

To identify regions suitable for Bayesian fine-mapping, we defined multi-trait QTL regions, similar to the method described by Zou et al. [35]. Briefly, a single-trait QTL region was defined as a region containing significant SNPs with distances between adjacent significant SNPs less than 500 kb and bounded by the final significant SNPs at both sides of the region. Subsequently, the overlapped single-trait QTL regions of different traits were merged and the merged regions were defined as the multi-trait QTL regions, which were subjected to further fine-mapping. If a QTL region contained only one significant SNP, we defined the tested fine-mapping region as within 100 kb of both sides of that SNP.

Then, a multi-trait Bayesian fine-mapping method, mvSuSiE [35], was used to identify putative causal variants within each region. mvSuSiE extends the Sum of Single Effects (SuSiE) model by performing multivariate analysis, which improves the power for identifying causal SNPs, and utilizes iterative Bayesian step-wise selection (IBSS) method to calculate cross-trait PIP that a SNP is causal for at least one trait. Based on these PIPs, mvSuSiE provides 95%-level credible sets (CS) of putative causal variants within each QTL region, where the sum of PIPs of the included SNPs is equal to or greater than 0.95. Additionally, the conditional local false sign rate (*lfsr*) [36] was calculated, defined as the posterior probability that the estimated effect of a SNP has the incorrect sign (±), given that the SNP has a nonzero effect. A small conditional *lfsr* of a SNP for a trait indicates high confidence in the sign of the effect. To evaluate the significance of the identified CS for each trait, mvSuSiE computes the average *lfsr*, calculated as a PIP-weighted average of the conditional *lfsr*’s for all SNPs in each CS. In this study, consistent with Zou et al. [35], when the average *lfsr* of a CS for a trait was less than 0.01, it was considered significant for that trait.

After fine-mapping, we estimated the heritability of the lead SNPs (SNPs with the largest PIP in each CS) for each trait,

4 $$ \begin{gathered} {\mathbf{y}} = {\mathbf{\mu }} + {\mathbf{Z}}_{{lead}} {\mathbf{a}}_{{lead}} + {\mathbf{Z}}_{{rest}} {\mathbf{a}}_{{rest}} + {\mathbf{e}}, \hfill \\ {\mathbf{a}}_{{lead}} \sim N\left( {0,{\mathbf{G}}_{{lead}} \sigma _{{{\text{a}}_{{lead}} }}^{2} } \right);{\mathbf{a}}_{{rest}} \sim N\left( {0,{\mathbf{G}}_{{rest}} \sigma _{{{\text{a}}_{{rest}} }}^{2} } \right);{\mathbf{e}}\sim N\left( {\mathbf{0},{\mathbf{I}}\sigma _{{\text{e}}}^{2} } \right) \hfill \\ \end{gathered} $$

where ${\mathbf{a}}_{lead}$ is the vector of additive genetic effects captured by all lead SNPs in the CSs that were significant for the given trait, ${\mathbf{a}}_{rest}$ is the vector of additive genetic effects captured by all rest SNPs not in these significant CSs. ${\mathbf{G}}_{lead}$ and ${\mathbf{G}}_{rest}$ are GRMs that are built using the lead SNPs and rest SNPs, respectively, which were constructed using the same method described in Eq. (1). ${\mathbf{Z}}_{lead}$ and ${\mathbf{Z}}_{rest}$ are the design matrices associating ${\mathbf{a}}_{lead}$ and ${\mathbf{a}}_{rest}$ with $\mathbf{y}$. The rest terms ($\mathbf{y}$, ${\varvec{\upmu}}$, $\mathbf{e}$, etc.) are the same as those in Eq. (1). The heritability of the lead SNPs was calculated as ${h}_{lead\_SNP}^{2}=\frac{{\sigma }_{{\text{a}}_{lead}}^{2}}{{\sigma }_{{\text{a}}_{lead}}^{2}+{\sigma }_{{\text{a}}_{rest}}^{2}+{\sigma }_{\text{e}}^{2}}$. Then, the proportion of the heritability explained by the lead SNPs was calculated as ${h}_{lead\_SNP}^{2}/{h}_{total\_SNP}^{2}$, where ${h}_{total\_SNP}^{2}$ was the heritability estimated using Eq. (1) based on GRM using whole genome-wide SNPs.

Additionally, we used SnpEff v5.2 [37] to annotate the types and genomic regions of all lead SNPs. For missense variants, the local structural characteristics of the wild type and mutant type proteins were assessed using DynaMut [38] to predict the thermodynamic stability (ability to keep its original structure and function) changes before and after the mutation. Additionally, we downloaded the ATAC-seq data of four cattle tissues (liver, lung, muscle, and mammary) from an organism-wide ATAC-seq peak catalog [39] to investigate whether the intergenic lead SNPs in the CSs overlapped with core and consensus segments called from ATAC-seq peaks. To obtain transcription factor (TF) information, FIMO v5.5.7 [40] was used to predict transcription factor binding sites on both wild type and mutant type sequences defined as ± 25 bp of lead SNP.

### Tissue specificity analysis

From the CattleGTEx database [21], we selected the tissues with sample size > 50 (22 tissues were selected) to identify tissue specificities with respect to the six milk-related traits using MAGMA v1.08 [41]. Firstly, we annotated all SNPs to genes based on the cattle genome annotation information using the “–annotate” function in MAGMA. We only extended 10 kb upstream and downstream of each gene to generate an annotation file as an input file. Then, we used the “SNPwise-mean” model in MAGMA leveraging the single-trait GWAS summary data (*P*-values) and the LD information between SNPs to perform gene-based association analysis, in which the SNP-based *P*-values were integrated to derive gene-based test statistics and corresponding *P*-values. Subsequently, we calculated the *P*-values of tissue specificity of all genes in the selected tissues based on the CattleGTEx data using the method described in Liu et al. [21], and selected the top 5% most specific genes for each tissue, which was defined as the tissue-specific gene sets (TSGS). Based on TSGS of all tissues, we generated a gene-set annotation file using the option “–set-annot” in MAGMA. Finally, we applied the “SNPwise-mean” model again to perform gene-set based association analysis between TSGS and the traits using the results of gene-based association analysis and the gene-set annotation file, in which the gene-based *P*-values were integrated to derive gene-set based *P*-values, and then the *P*-values for all TSGS were ranked across all tissues for each trait to obtain the most specific tissues associated with the traits studied.

### Mendelian randomization analysis

To identify putative functional genes for each trait, MR analysis were performed using gene expression levels as exposures and traits as outcomes using the single-trait GWAS summary data for all SNPs throughout the whole genome obtained in this study and the *cis*-eQTL summary data from CattleGTEx [21]. Based on the tissue specificity analysis, we focused on *cis*-eQTLs in four tissues, i.e., blood, mammary, milk cells, and liver. Firstly, we performed single-trait MR analysis using the PMR-Egger R package [42]. Furthermore, considering the possible pleiotropic associations of a gene with multiple traits, we also performed multi-trait MR analysis using the moPMR-Egger R package [43], which is an extension of PMR-Egger for multivariate MR analysis and tests causal effects of a gene on multiple traits jointly. To ensure that the selected IVs satisfied the core assumptions of MR, we used *cis*-eQTLs located within ± 500 kb of each gene and with association *P*-values < 5 × 10^−6^, to ensure strong and direct relationships with gene expression and thereby fulfilling the relevance assumption. To address the independence assumption and minimize confounding from correlated variants, LD clumping was performed by removing SNPs with *r*^2^ > 0.1, which ensured that the retained IVs were approximately independent and not tagging multiple signals. To satisfy the exclusion-restriction assumption, which requires that IVs affect the phenotype only through gene expression, the PMR-Egger and moPMR-Egger methods were employed to test and adjust for potential horizontal pleiotropy in the model. Gene-trait pairs that showed significant causal effects (FDR-adjusted *P*-values < 0.05) and no horizontal pleiotropy (*P*-values > 0.05) were regarded as significant causal associations.

### Colocalization analysis

We performed colocalization analysis using the COLOC R package (version 5) [44], with the runsusie and susie.coloc functions, to identify the shared causal variants between the milk-related traits and gene expressions in the four tissues mentioned above (blood, mammary, milk cells, and liver). COLOC uses the SuSiE regression framework to identify potential shared causal variants and provides posterior probabilities for five mutually exclusive hypotheses regarding the associations of variants with the trait and the expression of a gene: H0: no association with either the trait or the gene expression; H1: association with the trait only; H2: association with the gene expression only; H3: association with both the trait and the gene expression due to linkage of two distinct causal variants (one for the trait and the other for the gene expression); and H4: association with both the trait and gene expression due to a common single causal variant. A posterior probabilities value ≥ 0.8 for H4 was applied to define significant association.

## Results

### SNP-based heritabilies and genetic correlations

The estimated heritabilities and genetic correlations for the six milk-related traits are shown in Table 1. PP exhibited the highest heritability (${\widehat{h}}^{2}$ = 0.42 ± 0.01), followed by FP (${\widehat{h}}^{2}$ = 0.35 ± 0.02), and lowest was observed for SCS (${\widehat{h}}^{2}$ = 0.17 ± 0.01). The heritabilities of MY, FY and PY ranged from 0.19 to 0.29. The estimated genetic correlations ranged from strong positive (${\widehat{r}}_{g}$= 0.84 ± 0.01 for MY-PY, ${\widehat{r}}_{g}$= 0.67 ± 0.03 for MY-SCS, and ${\widehat{r}}_{g}$= 0.65 ± 0.02 for FP-PP), to medium positive or negative (${\widehat{r}}_{g}$ = 0.37 ± 0.04 for MY-FY, ${\widehat{r}}_{g}$ = − 0.34 ± 0.04 for FP-PY, and ${\widehat{r}}_{g}$ = − 0.11 ± 0.04 for PP-PY), to nearly zero (${\widehat{r}}_{g}$ = 0.06 ± 0.04 for PP-FY and ${\widehat{r}}_{g}$ = 0.03 ± 0.06 for FY-SCS), and to strong negative (${\widehat{r}}_{g}$ = − 0.61 ± 0.03 for MY-FP, ${\widehat{r}}_{g}=$− 0.58 ± 0.03 for MY-PP, and ${\widehat{r}}_{g}$= − 0.60 ± 0.03 for FP-SCS).

**Table 1:** The estimates of heritabilities (diagonal), and genetic correlations (below diagonal) and their SE for the six milk-related traits

Trait^1^	MY	FP	PP	FY	PY	SCS
MY	0.29 ± 0.01					
FP	− 0.61 ± 0.03	0.35 ± 0.02				
PP	− 0.58 ± 0.03	0.65 ± 0.02	0.42 ± 0.01			
FY	0.37 ± 0.04	0.43 ± 0.04	0.06 ± 0.04	0.20 ± 0.01		
PY	0.84 ± 0.01	− 0.34 ± 0.04	− 0.11 ± 0.04	0.56 ± 0.04	0.23 ± 0.01	
SCS	0.67 ± 0.03	− 0.60 ± 0.03	− 0.41 ± 0.04	0.03 ± 0.06	0.57 ± 0.04	0.17 ± 0.01

^1^MY, milk yield; FP, fat percentage; PP, protein percentage; FY, fat yield; PY, protein yield; SCS, somatic cell score

### GWAS and QTL regions

**Fig. 2:**
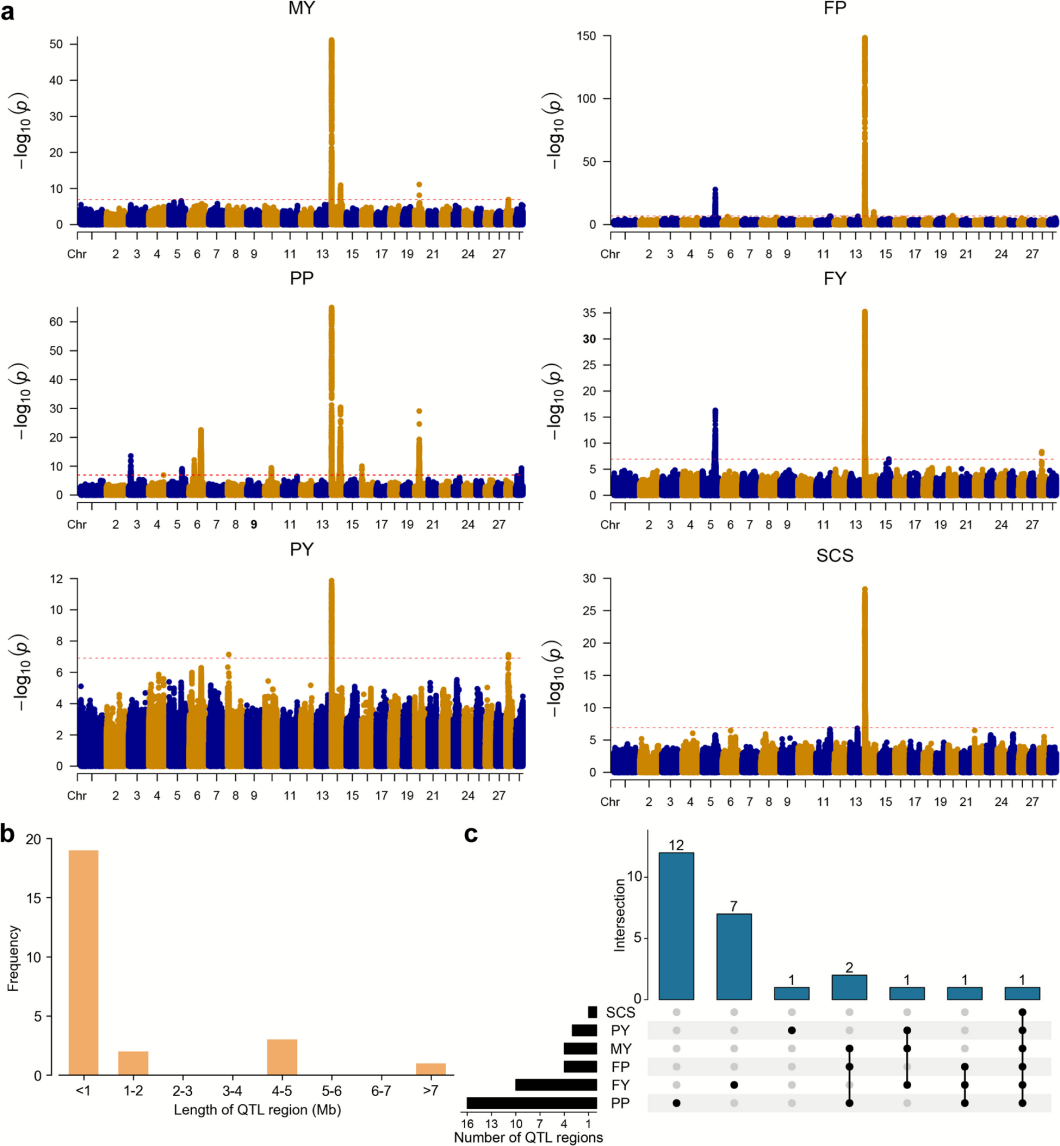
Summary of GWAS results. **a** Manhattan plot of GWAS analysis for six milk-related traits. The dotted red lines indicate the significance threshold (*P* = 1.2 × 10^−7^). **b** Frequency distribution of the length of QTL regions. **c** Numbers of overlapped QTL regions of different traits. MY: milk yield. FP: fat percentage. PP: protein percentage. FY: fat yield. PY: protein yield. SCS: somatic cell score

Using a genome-wide significance level of *P* < 1.2 × 10^−7^, we found 9688 significant SNPs for the six traits (MY, FP, PP, FY, PY, and SCS) (Fig. 2a). In total, 39 unique QTL regions associated with at least one of the six milk-related traits were detected, with the number of them ranging from only one for SCS to 16 for PP (see Additional file 2 Table S2). Then, we merged overlapping single-trait QTL regions into multi-trait QTL regions, remaining 25 QTL regions (see Additional file 2 Table S3). The sizes of the merged QTL regions ranged from 1 bp to 7.15 Mb with an average of 1.47 Mb. 76% of the intervals were less than 1 Mb in width (Fig. 2b). Most QTL regions were associated with only one trait (Fig. 2c), but four regions were associated with three traits and one region on chromosome 14 was associated with all six traits (see Additional file 2 Table S3).

### Multi-trait Bayesian fine-mapping for putative causal variants

**Fig. 3:**
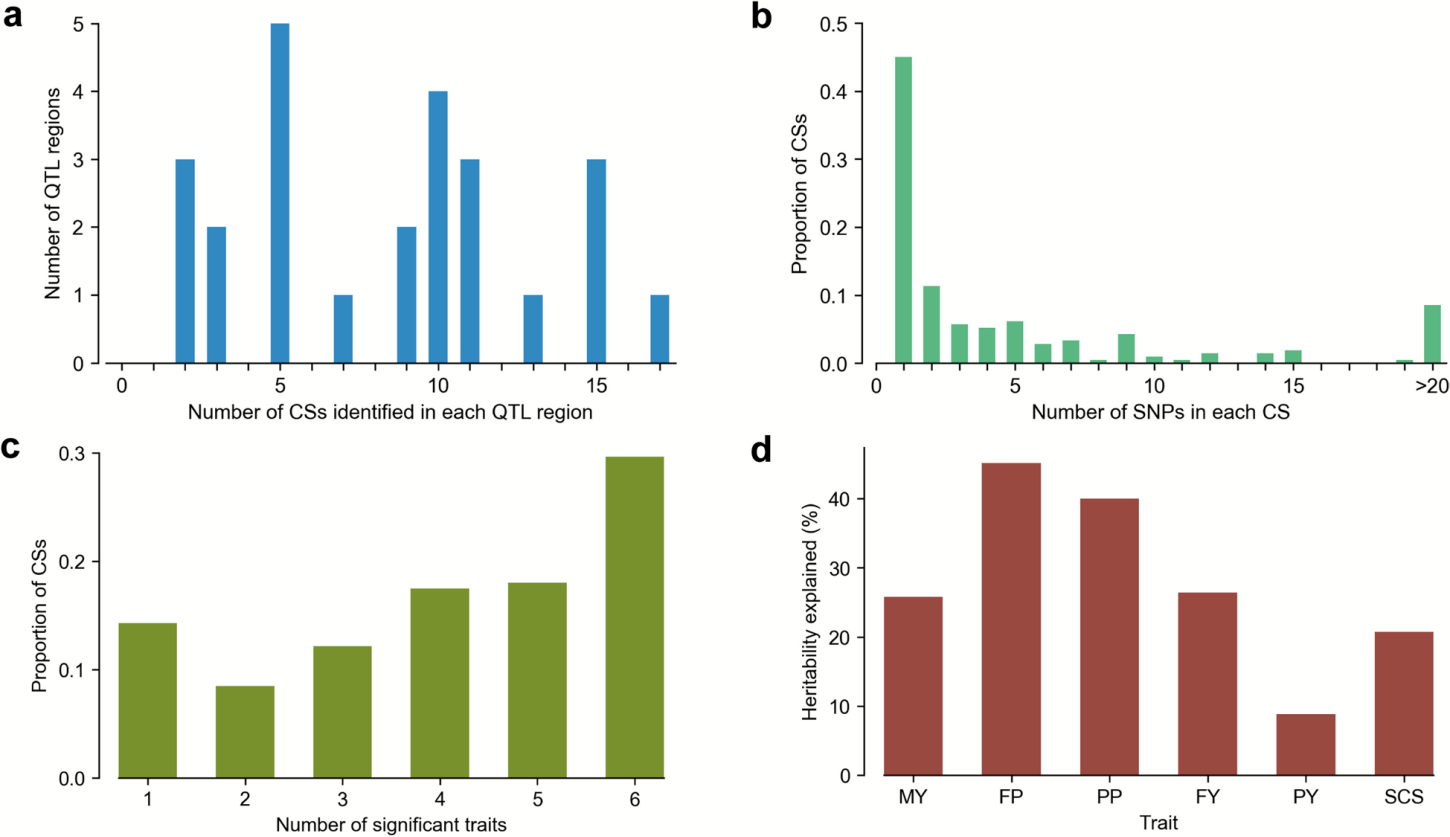
Summary of multi-trait Bayesian fine-mapping results. **a** Numbers of credible sets (CSs) of causal variants identified in each QTL region. **b** Numbers of SNPs in each CS. **c** Numbers of significantly associated traits (*average lfsr* < 0.01) in each CS. **d** Heritabilities explained by the lead SNPs in the significant CSs. MY: milk yield. FP: fat percentage. PP: protein percentage. FY: fat yield. PY: protein yield. SCS: somatic cell score

The multi-trait Bayesian fine-mapping within the 25 QTL regions identified 211 independent 95%-level CSs, with the minimum and maximum number of CSs in each QTL region being 2 and 17, respectively (Fig. 3a). The number of SNPs within each CS ranged from 1 to 233 with a median number of 2. About 45% of these CSs contained only one SNP (Fig. 3b). After filtering (average *lfsr* < 0.01), 189 CSs significantly associated with at least one trait were identified and approximately 30% of them were associated with all six traits (Fig. 3c). Additionally, we calculated the proportions of heritability explained by the lead SNPs in the significant CSs (Fig. 3d). The lead SNPs for four traits (MY, FP, PP, and FY) explained more than 25% of heritability. The largest proportion (45%) was observed for FP.

**Fig. 4:**
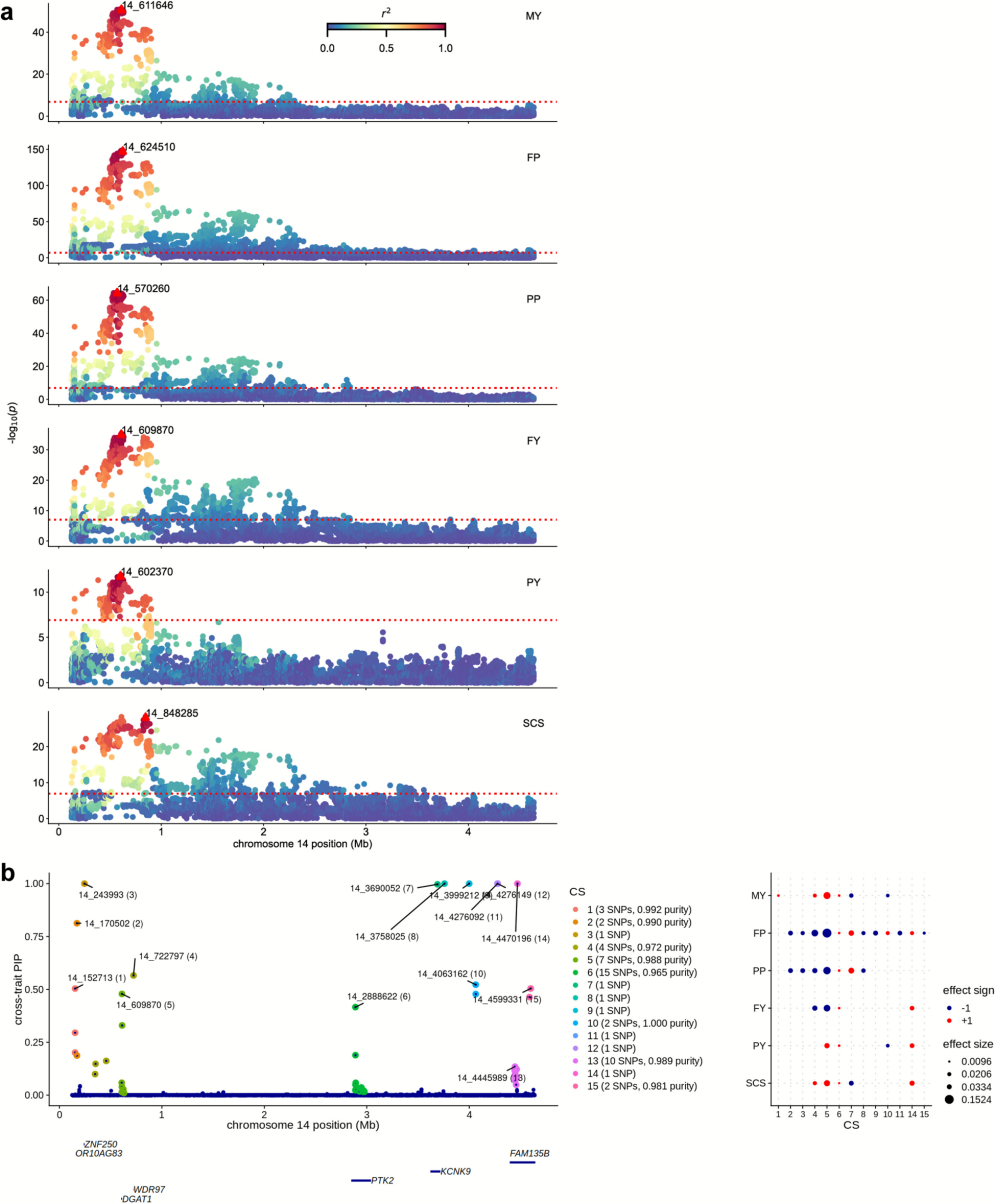
Local results of GWAS and multi-trait Bayesian fine-mapping in the region Chr14:125,070–4,637,446. **a** Local Manhattan plot of GWAS-results of six milk-related traits. The dotted red line indicates the significance threshold (*P* = 1.2 × 10^−7^). The red triangles represent the top (i.e., most significant) SNPs. The colors of the points indicate the strength of linkage disequilibrium (LD) of the top SNPs with other SNPs. **b** The results of multi-trait fine-mapping. The left-hand part shows the cross-trait posterior inclusion probabilities (PIPs) for each SNP in the QTL region. The labeled SNPs are the “lead SNPs”, i.e., SNPs with the highest cross-trait PIP in each CS. “Purity” is defined as the minimum absolute pairwise correlation (Pearson’s *r*) among SNPs in the CS. The related genes of the lead SNPs are displayed below. The right-hand part shows the posterior effect estimates of the sentinel SNPs whenever the CS is significant for the given trait (*average lfsr* < 0.01). MY: milk yield. FP: fat percentage. PP: protein percentage. FY: fat yield. PY: protein yield. SCS: somatic cell score

Chr14:12,570-4,637,446 is a well-known pleiotropic QTL region influencing multiple traits in dairy cattle. Our current GWAS revealed many pleiotropic significant signals within this region for the six traits (Fig. 4a). The 13 significant CSs within this region exhibited a wide variety of effect patterns (Fig. 4b), e.g., CS1, CS9, CS11, and CS15 were associated with only one trait, CS2, CS3, CS8, and CS10 with two or three milk production traits, and CS5 and CS6 with all the six traits.

### Annotation of lead SNPs in the credible sets

**Fig. 5:**
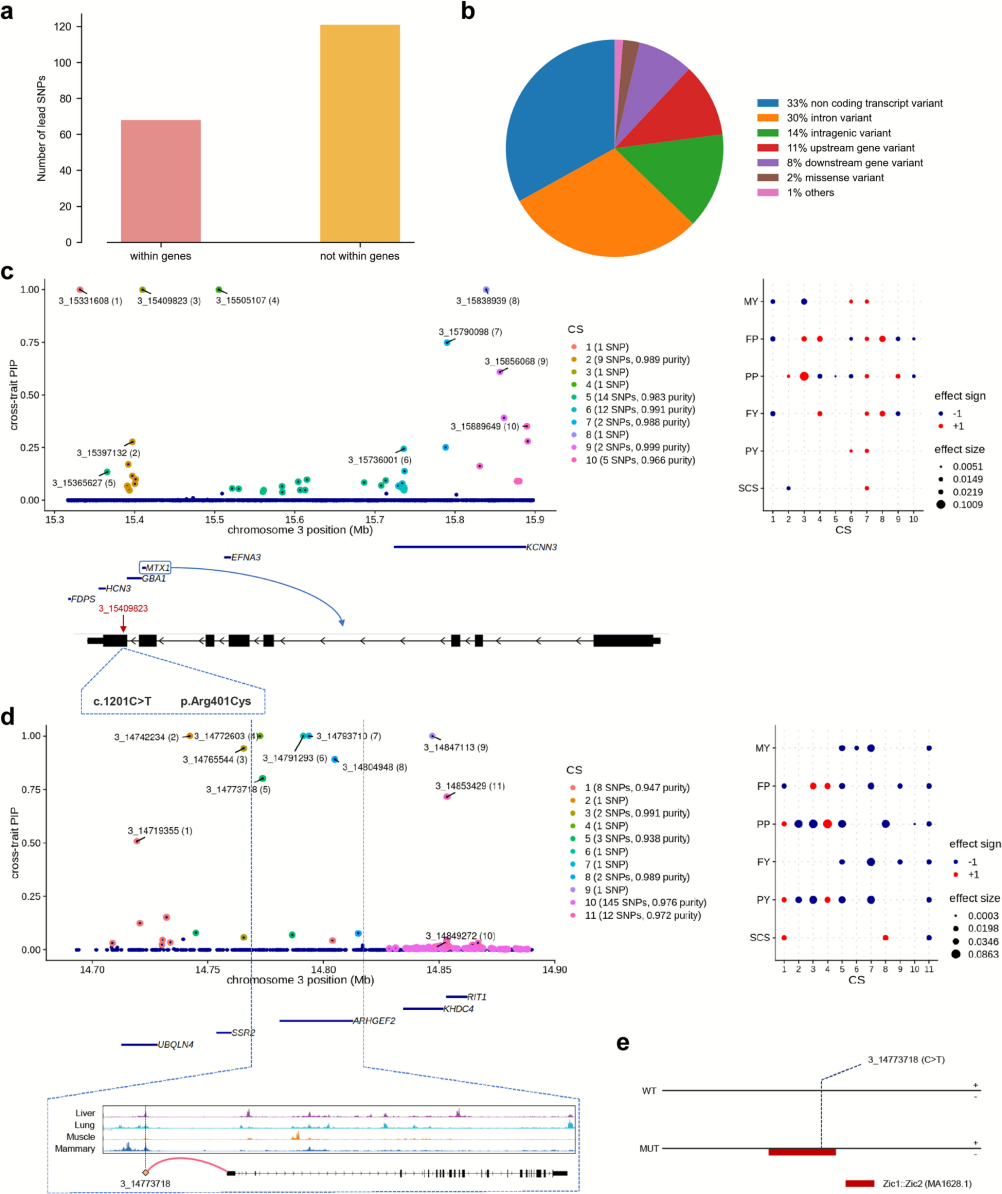
Summary of annotations of the lead SNPs in the CSs. **a** Numbers of the lead SNPs within genes or not within genes. **b** Proportions of different categories to which the lead SNPs are annotated. **c** Fine-mapping results for the QTL region Chr3:15,316,655–15,897,327 containing the lead SNP 3_15409823 which is a missense variant within *MTX1* (c.1201C > T, p.Arg401Cys). **d** Fine-mapping results for the QTL region Chr3:14,690,087–14,890,087 containing the lead SNP 3_14773718 which is coincided with ATAC-seq peaks observed in liver, lung, and mammary tissues. **e** Predicted transcription factor (TF) binding changes caused by SNP 3_14773718 (C > T). The reference allele (WT) and alternative allele (MUT) sequences are shown with predicted TF binding regions. MY: milk yield. FP: fat percentage. PP: protein percentage. FY: fat yield. PY: protein yield. SCS: somatic cell score

We used SnpEff [37] to annotated the 189 lead SNPs in the significant CSs. Of these lead SNPs, 68 (36%) are located within protein-coding genes (Fig. 5a), 62 (33%) are non-coding transcript variants, 57 (30%) are intron variants, and 3 (2%) are missense variants (Fig. 5b). Of the three missense variants, the first one (3_15409823) within *MTX1* on chromosome 3 results in an Arginine (Arg) to Cysteine (Cys) substitution at position 401 of the MTX1 protein (Fig. 5c). The second one (20_31888449) within *GHR* on chromosome 20 results in a Phenylalanine (Phe) to Tyrosine (Tyr) substitution at position 257 of the GHR protein (see Additional file 1 Figure S5a). The CSs harboring either of the two variants exhibited significant effects on MY, FP, and PP. The third one (29_40892385) within *AHNAK* on chromosome 29 results in a Serine (Ser) to Asparagine (Asn) substitution at position 1140 of AHNAK protein with the significant effect on FP (see Additional file 1 Figure S5b).

As mentioned above, most lead SNPs are located in non-coding or intergenic regions, implying they may function as regulatory variants. To investigate their regulatory potential, we downloaded the ATAC-seq data of four tissues (liver, lung, muscle, and mammary) from the organism-wide ATAC-seq peak catalog [39]. As expected, we found that some lead SNPs were located in the ATAC-seq peaks. For example, we identified 11 CSs within the region Chr3:14,690,087–14,890,087. The lead SNP (3_14773718) in CS5, located at 7742 bp upstream of the *ARHGEF2* gene and showed significant association with all five milk production traits, was coincided with ATAC-seq peaks observed in liver, lung, and mammary tissues (Fig. 5d). Similarly, in the region Chr16:1,786,132–1,791,556, we identified a lead SNP (16_1790694) in CS2 which is overlapped with ATAC-seq peaks in lung, muscle, and mammary tissues (see Additional file 1 Figure S7a). This SNP, located 109 kb upstream of the *SOX13* gene, exhibited significant associations with MY, FP, and PP.

### Tissue specificity analysis for tissue-trait associations

Among the 22 tissues with more than 50 samples, we found that milk cells were the most specific tissue for MY, FY, and PY. In addition, milk cells were the third specific tissue for PP, after rumen and lung (see Additional file 1 Figure S9). The mammary tissue was relatively specific for several traits, especially for MY and FP. Blood was closely related to several traits, ranking among the top five tissues for MY, FP, FY, and PY. For SCS, leukocytes emerged was the most related tissue (see Additional file 1 Figure S9).

### Mendelian randomization to identify putative causal genes

**Fig. 6:**
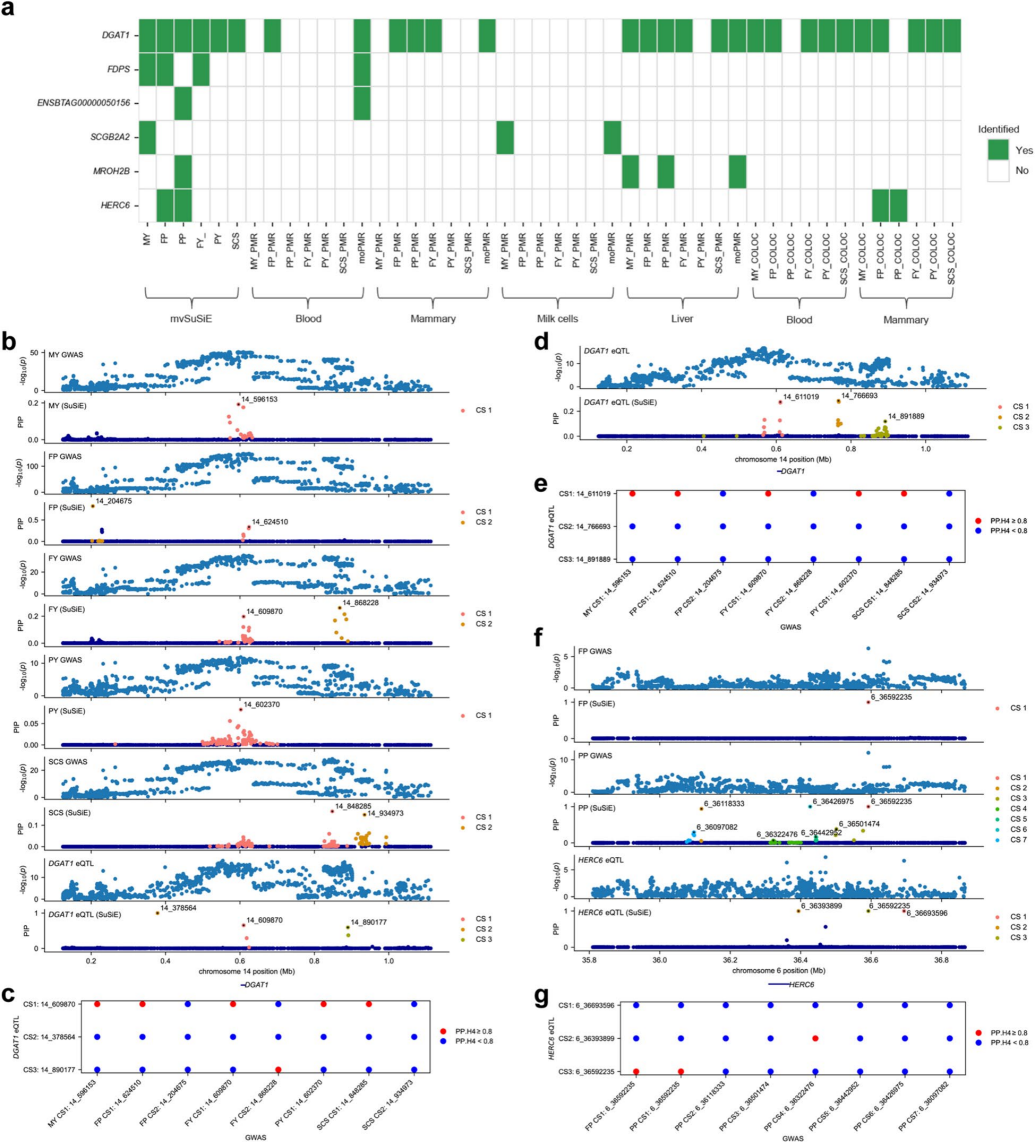
Summary of the Mendelian randomization (MR) and colocalization analysis. **a** Overlapped genes identified by multi-trait Bayesian fine-mapping, MR, and colocalization analysis. MR and colocalization were performed using GWAS summary data and *cis*-eQTL summary data for four tissues (blood, mammary, milk cells, and liver). **b** Colocalization between GWAS of five traits (MY, FP, FY, PY and SCS) and the blood *cis*-eQTL of *DGAT1*. The labeled SNPs are the “lead SNPs” in each CS. **c** The shared causal variants identified by fine mapping for MY, FP, FY, PY, and SCS with the blood *cis*-eQTL of *DGAT1* on Chr14. The significant shared variants are highlighted in red (posterior probability for H4, PP.H4 ≥ 0.8). **d** eQTL mapping and fine-mapping (SuSiE) of *DGAT1* in mammary on Chr14. **e** The shared causal variants identified by fine mapping for MY, FP, FY, PY, and SCS with the mammary *cis*-eQTL of *DGAT1* on Chr14. **f** Colocalization of GWAS and fine-mapping (SuSiE) results of FP and PP and the blood *cis*-eQTL of *HERC6* on Chr6. **g** The shared causal variants identified by fine mapping for MY, FP, FY, PY, and SCS with the blood *cis*-eQTL of *HERC6*. MY: milk yield. FP: fat percentage. PP: protein percentage. FY: fat yield. PY: protein yield. SCS: somatic cell score

Leveraging the GWAS summary data from this study and the *cis*-eQTL summary data from CattleGTEx [21], we used PMR-Egger (single trait MR) [42] and moPMR-Egger (multi-trait MR) [43] to identify putative causal genes underlying the six milk-related traits. In above, we observed specific associations of gene expressions in blood, mammary, and milk cells with the investigated milk-related traits. Additionally, it is well known the liver also plays an important role in regulating milk production traits [45, 46]. Therefore, we selected the *cis*-eQTL data from these four tissues for both PMR-Egger (see Additional file 1 Figs. S10–S13) and moPMR-Egger analysis (see Additional file 1 Figure S14). We identified 268 genes exhibiting significant causal effects on at least one of the six traits (see Additional file 2 Tables S5–S8), five of which (*DGAT1*, *FDPS*, *ENSBTAG00000050156*, *SCGB2A2*, and *MROH2B*) were overlapped with the genes identified through multi-trait fine-mapping (Fig. 6a). Notably, *DGAT1* exhibited significant effects on all traits in the fine-mapping results and its expression in mammary and liver showed causal effects on multi-traits in the MR analysis.

#### Colocalization analysis to identify shared putative causal variants for the milk-related traits and gene expressions

Through colocalization analysis, we identified two putative causal *cis*-eQTLs of *DGAT1* in blood (Fig. 6b) which were significantly colocalized with some putative causal GWAS signals (posterior probability for H4 > 0.8), one (14_609870) with GWAS signals for five traits (MY, FY, FP, PY, and SCS) and the other (14_890177) with one GWAS signal for FY (Fig. 6c). Similarly, one putative causal *cis*-eQTLs of *DGAT1* in mammary (14_611019) was colocalized with the same five GWAS signals as the one in blood (14_609870) (Fig. 6d and e). In addition, two putative causal *cis*-eQTLs of *HERC6* in mammary, 6_36393899 and 6_36592235, were colocalized with some GWAS signals, one with GWAS signal for PP and the other with GWAS signals for FP and PP (Fig. 6f and g).

## Discussion

In this study, we performed GWAS for six milk-related traits using imputed sequence data in a Chinese Holstein population of 11,059 cows. Based on the GWAS results, we identified 25 unique QTL regions associated with one or multiple traits. The subsequent Bayesian fine-mapping analysis within these regions discovered 189 significant CSs containing at least one putative causal SNP. Notably, multiple CSs were identified within all QTL regions. In fact, both SuSiE (single trait fine mapping) [10] and mvSuSiE (multi-trait fine mapping) [35] allow a QTL region to contain multiple CSs. In the former case, multiple CSs may be detected in a QTL region for a single trait, while in the latter case, multiple CSs may be detected in a QTL region for multiple traits (as well as for a single trait). Several studies applied SuSiE (e.g., in human [47] and in cattle [16]) and identified multiple CSs in some QTL regions. mvSuSiE extends SuSiE by performing multivariate analysis with the aim to improve the power of fine-mapping. Multiple causal variants within a QTL region are common and biologically plausible. We also calculated the LD between the lead SNPs of CSs within each QTL region (see Additional file 2 Table S4). The majority of the pair-wise LDs were very low (*r*^2^ < 0.1). For example, for the lead SNPs in the DGAT1 region (14:125,070–4,637,446), among the 78 lead SNP pairs, there were only five pairs showing *r*^2^ > 0.1, supporting the presence of multiple independent association signals within the QTL region. The number of SNPs in each CS distributed from 1 to 233 with a median of two. The multiple SNPs in a CS are usually highly correlated with each other. For example, in CS6 in the QTL region Chr6:81,704,992–88,851,462, which included 233 SNPs, the “Purity” of these SNPs, defined as the minimum absolute pairwise correlation (Pearson’s r) among SNPs in a CS in mvSuSiE, was 0.859 (see Additional file 1 Figure S2). This high level of correlation makes it difficult to confidently assign high PIPs to SNPs. Therefore, moderate PIPs were distributed across many highly correlated variants. Although such CSs with a large number of SNPs were identified, they are generally not prioritized in downstream analysis due to their low resolution. In addition, we found that different CSs within the same QTL region exhibited distinct “effect patterns”, with each CS associated with different traits. The different “effect patterns” are partly due to the differences in statistical power across traits. Differences in genetic architecture of difference traits, e.g., the pleiotropy of the causal variants, may also result in different effect patterns.

Within the CSs identified, there are several causal variants reported in previous studies. In particular, a CS (including 7 significant SNPs) in the *DGAT1* region was identified to be strongly associated with all the six traits, and the lead SNP (14_609870) in this CS has been reported to be highly significant for these traits in many GWAS studies [48–50]. It should be noted that the well-known missense variant 14_611019 (or 14_611020) (K232A) in *DGAT1*, which have been proven to be functional mutations for milk production traits [51], are very close to this lead SNP, and are also included in this CS. The possible reason that we identified 14_609870 as the lead SNP, instead of 14_611019 (or 14_611020), is that 14_609870 is included in the three chips, while 14_611019 and 14_611020 are not and their genotypes were referred by imputation. Therefore, the genotypes of 14_609870 had higher accuracy than the imputed ones. The QTL region on chromosome 6 (Chr6:81,704,992–88,851,462) spans 7.15 Mb and contains 17 CSs, including several previously reported candidate genes for milk-related traits. This region overlaps some casein genes (e.g., *CSN1S1*, *CSN1S2*, and *CSN3*)*,* which has been repeatedly reported to be candidate genes for milk production traits [52]. There were several putative causal SNPs identified for these genes, such as 6_85393113 in CS5 for *CSN1S1*, 6_85530762 in CS7 for *CSN1S2*, and 6_85664195 in CS8 for *CSN3* (see Additional file 2 Table S3). In contrast, we did not detect any putative causal variants for *GC*, previously reported as a candidate gene for clinical mastitis [53, 54]. Lee et al. [55] found a ~ 12 kb copy number variant (CNV) encompassing a regulatory enhancer of *GC* which was associated with clinical mastitis. Since our fine-mapping analysis was based on SNP data, it was not able to directly capture the associations of structural variants such as CNVs. We also checked the lead SNP (rs110813063, 6_86951401) for SCS, which reported to be in strong LD with the 12 kb CNV by Lee et al. [55]. This SNP showed a weak association with SCS in our study (*P*-value = 0.0036, MAF = 0.45). It is uncertain whether this SNP has causal effect on SCS in our population.

Among the lead SNPs in the significant CSs, three missense variants within protein-coding genes were discovered, i.e., 3_15409823 (p.Arg401Cys) within *MTX1* with effects on MY, FP and PP, 20_31888449 (p.Phe257Tyr) within *GHR* with effects on MY, FP and PP, and 29_40892385 (p.Ser1140Asn) within *AHNAK* with effect on FP. *GHR* is a well-known candidate gene for milk production traits [56, 57], and the missense variant 20_31888449 (p.Phe257Tyr) has been proposed as a putative causal variant in *GHR* [58]. *MTX1* has been reported as a candidate gene for PP [59] and milk lactose percentage [60]. In contrast, no previous studies have reported an association between AHNAK and milk production traits in dairy cattle. AHNAK is a large protein that acts on nucleoproteins to regulate a wide variety of biological functions, such as adipogenesis [61], browning [62], and adipocyte differentiation [63]. To explore the potential mechanisms underlying the effects of these missense variants, we predicted the protein stability changes upon these missense mutations using DynaMut [38]. Since the 3D structure of the AHNAK protein is not available in the AlphaFold database [64], we only analyzed the MTX1 and GHR proteins (see Additional file 1 Figure S6). Structurally, the atomic contacts and bonds in a protein can be disrupted by missense mutations, both are referred as “destabilizing”, which can lead to a change in molecule flexibility (measured by vibrational entropy energy) of the protein. We found that the missense mutation in *MTX1* caused an increase in molecule flexibility of the MTX1 protein (see Additional file 1 Figures S6a and b), while that in *GHR* caused a decrease in molecular flexibility of the GHR protein (see Additional file 1 Figures S6c and d). The molecule flexibility is essential for a protein to effectively perform its function, such as responding to environmental changes, ligand binding, and chemical modifications [65], and therefore a perturbation that changes the flexibility of a protein may interfere with its function.

In addition to the protein coding variants, there are 68 non-coding regulatory variants among the lead SNPs, implying they may have some regulatory functions for the traits of interest. To investigate such functions, we downloaded the ATAC-seq data of four tissues (liver, lung, muscle, and mammary) from an organism-wide ATAC-seq peak catalog [39], as the ATAC-seq peaks indicate the open chromatin regions which may contain regulatory elements influencing gene expression. As expected, several lead SNPs overlapped with ATAC-seq peaks, suggesting potential regulatory activity. In particular, the lead SNP (3_14773718) of CS5, located at 7,742 bp upstream of *ARHGEF2* and significantly associated with all five milk production traits, is coincided with ATAC-seq peaks in liver, lung, and mammary tissues (Fig. 5d). We predicted the change in transcription factor (TF) binding to *ARHGEF2* resulted by this mutation (C > T) and found that the TF Zic1::Zic2 could bind to the mutant type but not to the wild type (Fig. 5e). Similarly, the lead SNP (16_1790694) of CS2, located at 109 kb upstream of *SOX13* and significantly associated with MY, FP, and PP, overlapped with ATAC-seq peaks in lung, muscle, and mammary tissues (see Additional file 1 Figure S7a). *SOX13* was also identified as a candidate gene for MY by Krizanac et al. [66]. TF prediction revealed that the mutant type (A > G) causes loss of binding for two TFs (MAFG::NFE2L1 and SOX2), but gain of binding for two new TFs (Bach1::Mafk and ZNF701) (see Additional file 1 Figure S7b). Obviously, these changes in TF binding will lead to the changes of expressions of these genes in the relevant tissues.

Our MR analysis identified 268 genes whose expressions had predicted causal effects on one or multiple milk-related traits. But only five of them, i.e., *DGAT1*, *FDPS*, *ENSBTAG00000050156*, *SCGB2A2*, and *MROH2B*, were also identified by the multi-trait fine-mapping. It should be noticed that the consistency between the two approaches is very low. One reason for this low consistency is that not all causal genes exert their effects on traits via their expressions [67]. Another possible reason is that the *cis*-eQTL data from CattleGTEx we used for MR analysis was based on gene expression data from many different studies with high heterogeneity in breed, age, and environment involved in these studies, and the sample sizes for most tissues were rather small, although we selected tissues with sample sizes larger than 50, and the four finally focused tissues (blood, mammary, milk cells, and liver) had sample sizes of 698, 175, 173, and 576, respectively, which may result in high false positive rate and low power in eQTL identification. Among these five overlapping genes, the previously repeated reported causal associations between *DGAT1* and milk-related traits were further confirmed in both fine-mapping and MR analysis. *FDPS*, associated with MY, FP, and FY, is involved in cholesterol biosynthesis [68]. Given the essential role of cholesterol in fatty acid and lipid metabolism in cattle [69], *FDPS* may influence milk fat synthesis through its function in this pathway. *ENSBTAG00000050156* is a protein coding gene with unknown function and annotation. *SCGB2A2*, identified as a candidate causal gene for MY in both fine-mapping and MR analysis, is a breast-specific member of the SCGB gene family and a marker for breast cancer in human [70]. *MROH2B* was identified as a candidate causal gene for PP. However, study on *MROH2B* is scarce, and its biological function remains largely uncharacterized.

Through colocalization analysis, we identified a few variants which exhibited significant common causal associations with one or multiple milk-related traits as well as with expressions of some genes. In particular, the SNPs 14_609870 and 14_611019 within *DGAT1*, which were found to be the causal *cis*-eQTLs for the expression of *DGAT1* in blood and mammary, respectively, were colocalized with the causal variants for MY, FP, FY, PY, and SCS. 14_611019 is a missense variant, which is a well-known causal variant for milk production traits due to its influence on activity and specificity of DGAT1 enzyme [71]. Here, we confirmed that this variant is also a putative causal variant for the expression of *DGAT1* in mammary. Two putative causal *cis*-eQTLs (6_36393899 and 6_36592235) for *HERC6* in mammary were colocalized with FP and PP. *HERC6* has been reported as a candidate gene for crude protein percentage [4] and lactation persistency [72] in cattle.

## Conclusions

In summary, we performed GWAS for six milk-related traits using imputed sequence data in a Chinese Holstein population and identified 25 QTL regions. Within these regions, we fine-mapped 189 significant independent CSs containing at least one putative causal variant for one or multiple milk-related traits. Through integrative analysis of Bayesian fine-mapping, MR, and colocalization, we prioritized a number of causal genes, including the well-known *DGAT1* and *GHR* genes and several novel genes, including *AHNAK*, *ARHGEF2*, *SOX13*, *FDPS*, *SCGB2A2*, and *MROH2B*. Our findings contribute to the existing understanding of genetic architecture of milk-related traits and highlight the effectiveness of our analysis strategy.

## Supplementary Information

Additional file1 (DOCX 4125 KB)

Additional file2 (XLSX 81 KB)

## Data Availability

Genotype data and trait data for dairy cattle used in this study are the property of the dairy farmers of China, and thus are not publicly available.
