# Supplementary material for: Integrative analysis of GWAS, Bayesian fine-mapping, Mendelian randomization and colocalization reveals genetic determinants underlying milk-related traits in dairy cattle
Source: Genet Sel Evol. 2026 Jan 3;58:4. doi: 10.1186/s12711-025-01028-3 (PMC12784616; doi:10.1186/s12711-025-01028-3)
Supplement: Supplementary file 1 — Additional file1 (DOCX 4125 KB) [file 12711_2025_1028_MOESM1_ESM.docx]

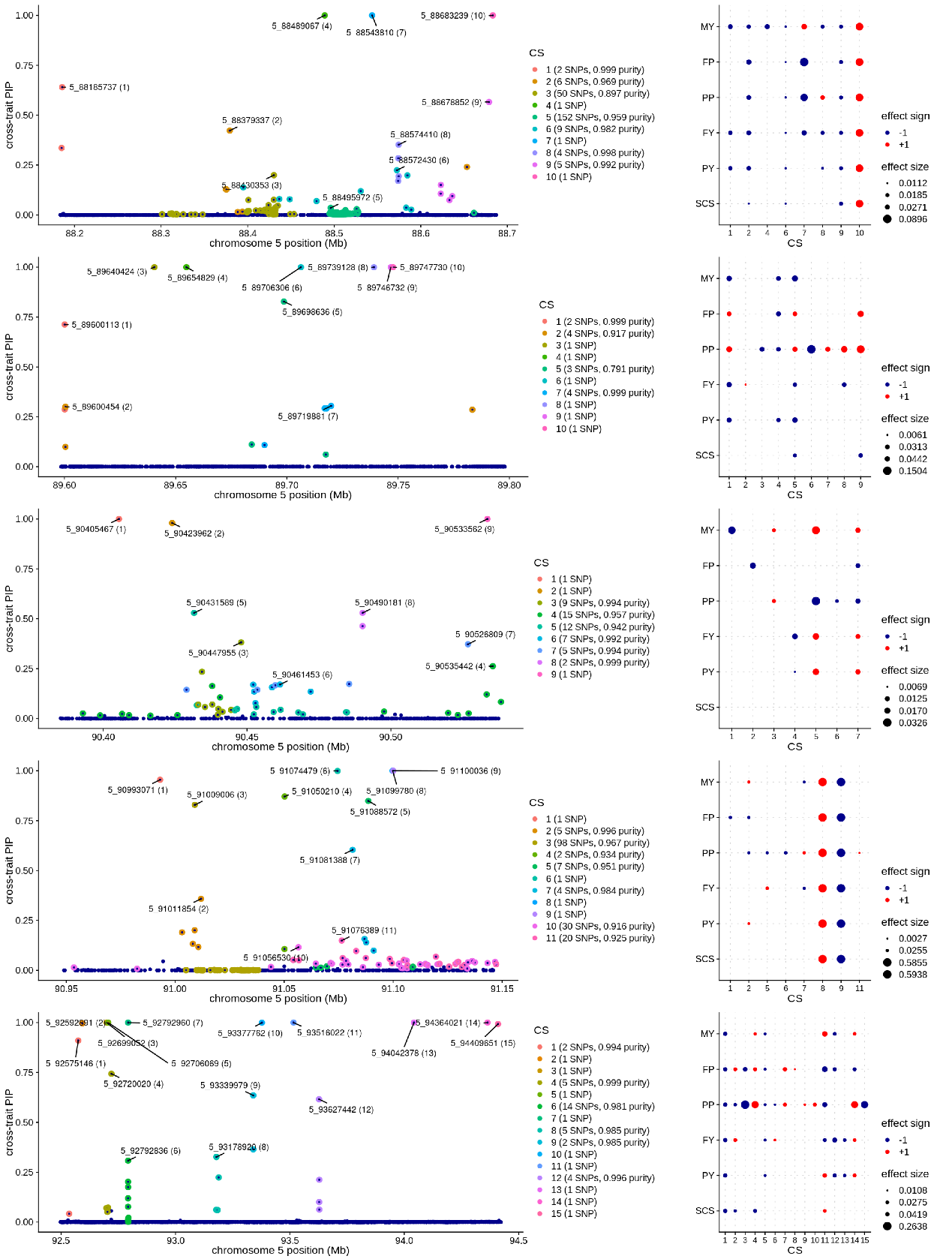


**Figure S1** **Results of multi-trait Bayesian fine-mapping on chromosome 5.** The left-hand part shows the cross-trait posterior inclusion probabilities (PIPs) for each SNP in the QTL region. The labeled SNPs are the “lead SNPs”, i.e., SNPs with the highest cross-trait PIP in each CS. “Purity” is defined as the minimum absolute pairwise correlation (Pearson’s *r*) among SNPs in the CS. The right-hand part shows the posterior effect estimates of the sentinel SNPs whenever the CS is significant for the given trait (*average lfsr* < 0.01). MY: milk yield. FP: fat percentage. PP: protein percentage. FY: fat yield. PY: protein yield. SCS: somatic cell score.


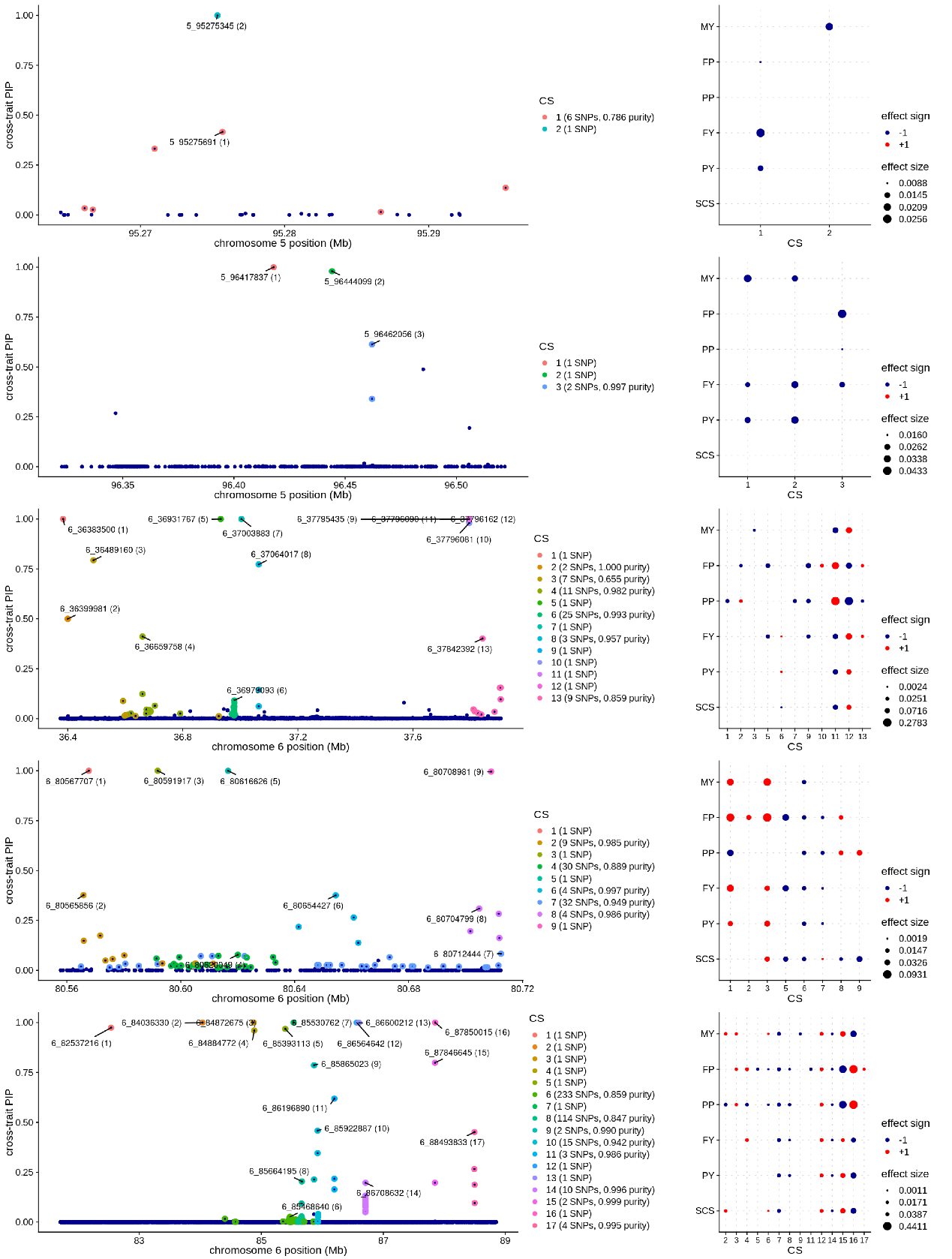


**Figure S2** **Results of multi-trait Bayesian fine-mapping on chromosomes 5 and 6.** The left-hand part shows the cross-trait posterior inclusion probabilities (PIPs) for each SNP in the QTL region. The labeled SNPs are the “lead SNPs”, i.e., SNPs with the highest cross-trait PIP in each CS. “Purity” is defined as the minimum absolute pairwise correlation (Pearson’s *r*) among SNPs in the CS. The right-hand part shows the posterior effect estimates of the sentinel SNPs whenever the CS is significant for the given trait (*average lfsr* < 0.01). MY: milk yield. FP: fat percentage. PP: protein percentage. FY: fat yield. PY: protein yield. SCS: somatic cell score.


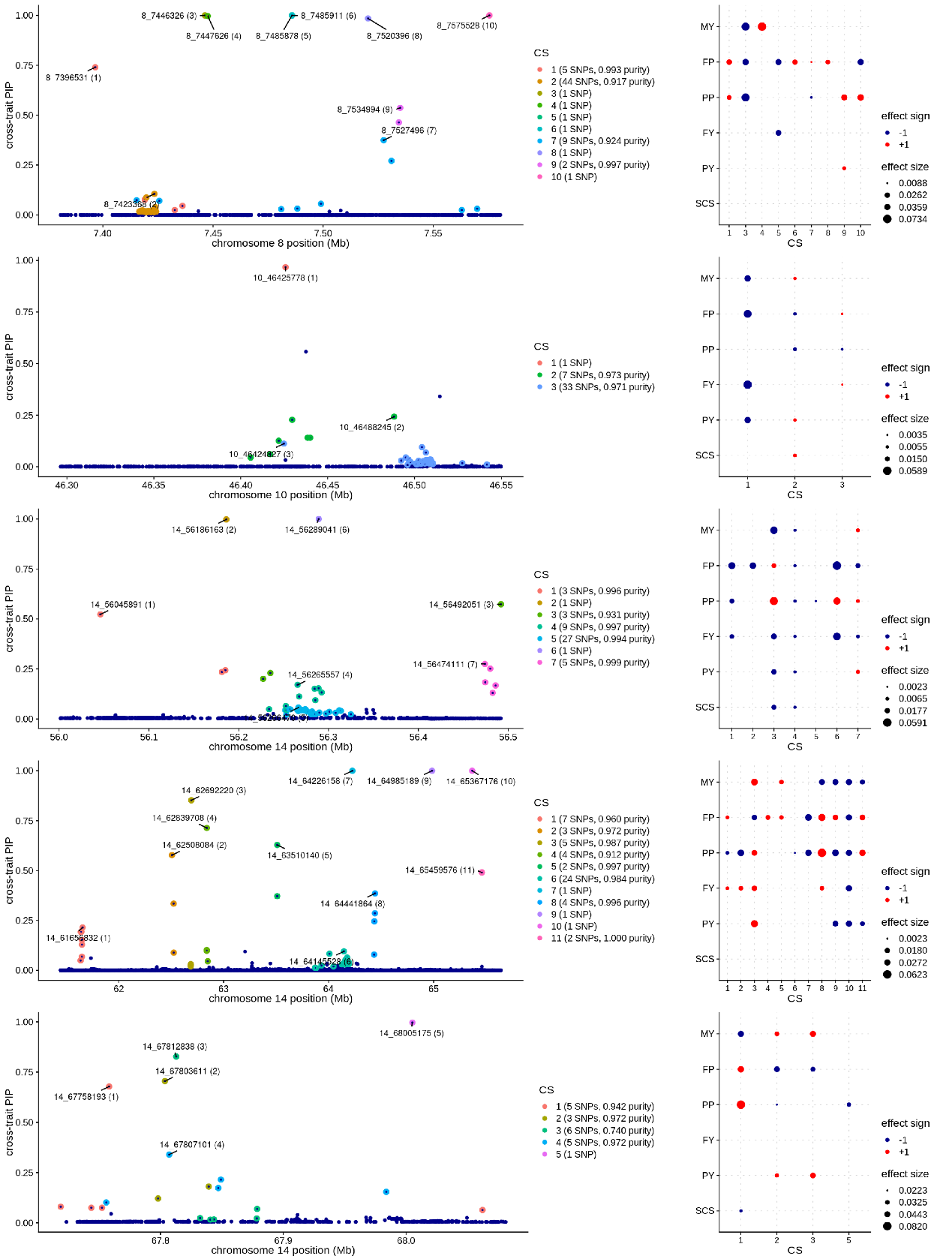


**Figure S3** R**esults of multi-trait Bayesian fine-mapping on chromosomes 8, 10 and 14.** The left-hand part shows the cross-trait posterior inclusion probabilities (PIPs) for each SNP in the QTL region. The labeled SNPs are the “lead SNPs”, i.e., SNPs with the highest cross-trait PIP in each CS. “Purity” is defined as the minimum absolute pairwise correlation (Pearson’s *r*) among SNPs in the CS. The right-hand part shows the posterior effect estimates of the sentinel SNPs whenever the CS is significant for the given trait (*average lfsr* < 0.01). MY: milk yield. FP: fat percentage. PP: protein percentage. FY: fat yield. PY: protein yield. SCS: somatic cell score.


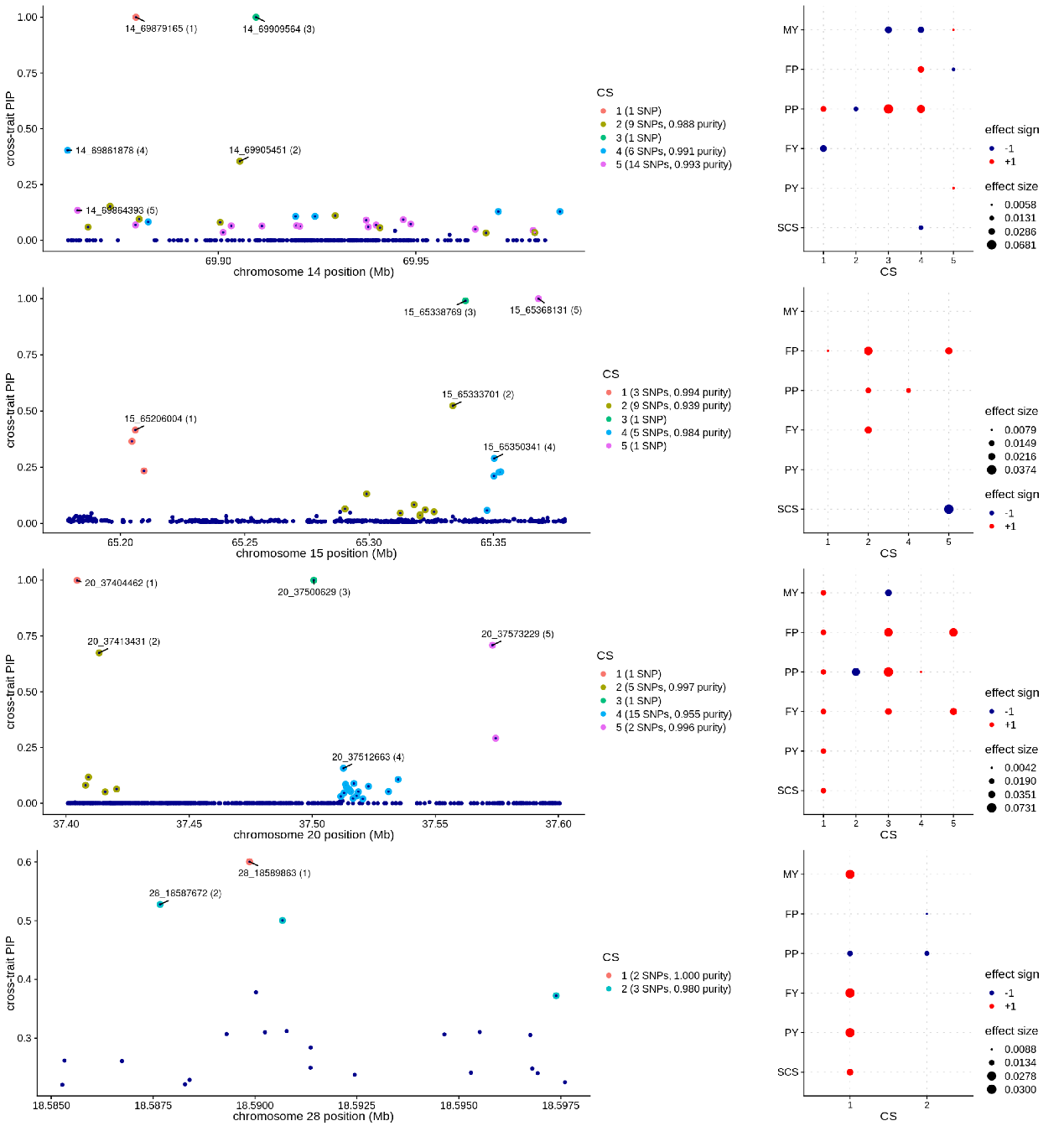


**Figure S4** **Results of multi-trait Bayesian fine-mapping on chromosomes 14, 15, 20 and 28.** The left-hand part shows the cross-trait posterior inclusion probabilities (PIPs) for each SNP in the QTL region. The labeled SNPs are the “lead SNPs”, i.e., SNPs with the highest cross-trait PIP in each CS. “Purity” is defined as the minimum absolute pairwise correlation (Pearson’s *r*) among SNPs in the CS. The right-hand part shows the posterior effect estimates of the sentinel SNPs whenever the CS is significant for the given trait (*average lfsr* < 0.01). MY: milk yield. FP: fat percentage. PP: protein percentage. FY: fat yield. PY: protein yield. SCS: somatic cell score.


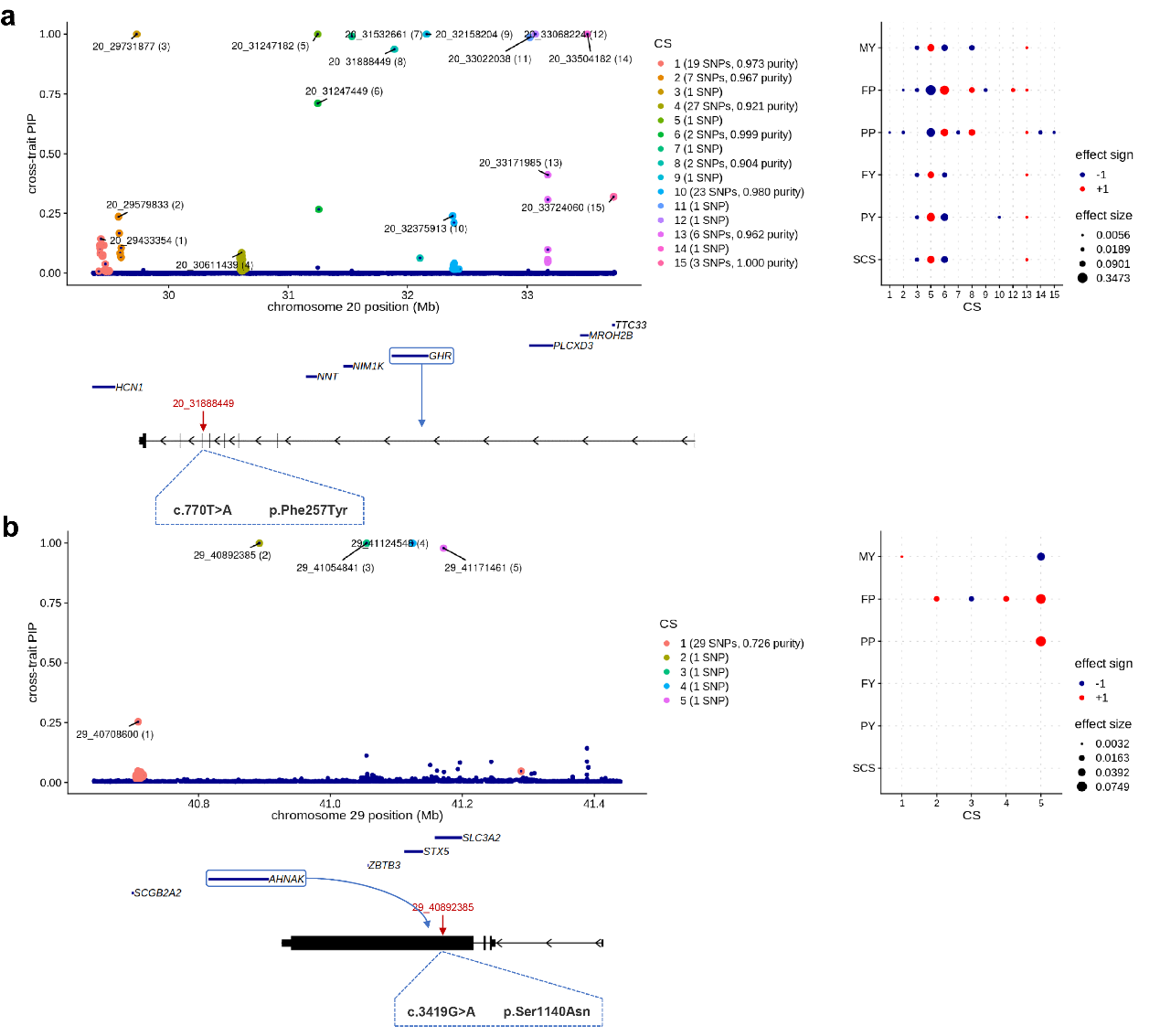


**Figure S5** **Results of multi-trait Bayesian fine-mapping in the QTL region Chr20:29,367,221-33,732,180 and Chr29:40,641,719-41,439,204.** (**a**) Fine-mapping results for the QTL region Chr20:29,367,221-33,732,180 containing the lead SNP 20_31888449 which is a missense variant within *GHR* (c.770T>A, p.Phe257Tyr). (**b**) Fine-mapping results for the QTL region Chr29:40,641,719-41,439,204 containing the lead SNP 29_40892385 which is a missense variant within *AHNAK* (c.3419G>A, p.Ser1140Asn). MY: milk yield. FP: fat percentage. PP: protein percentage. FY: fat yield. PY: protein yield. SCS: somatic cell score.


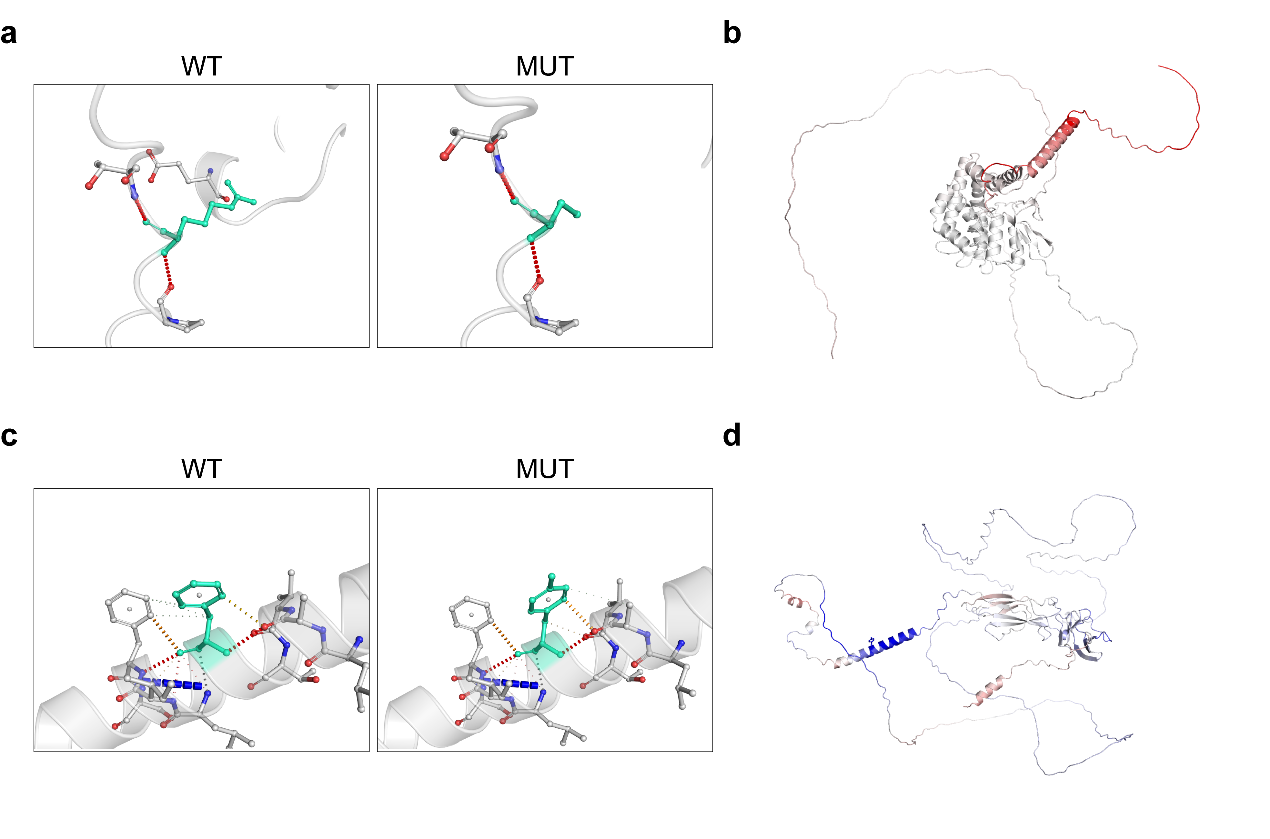


**Figure S6** **Results of DynaMut analysis.** Prediction of interatomic interactions wild type and mutant type of MTX1 protein (**a**) and GHR protein (**c**). Wild type and mutant type are colored in light-green and are also represented as sticks alongside with the surrounding residues which are involved on any type of interactions. Δ Vibrational Entropy Energy between wild type and mutant type of MTX1 protein (**b**) and GHR protein (**d**). Amino acids colored according to the vibrational entropy change upon mutation. Blue represents a rigidification of the structure and red a gain in flexibility.


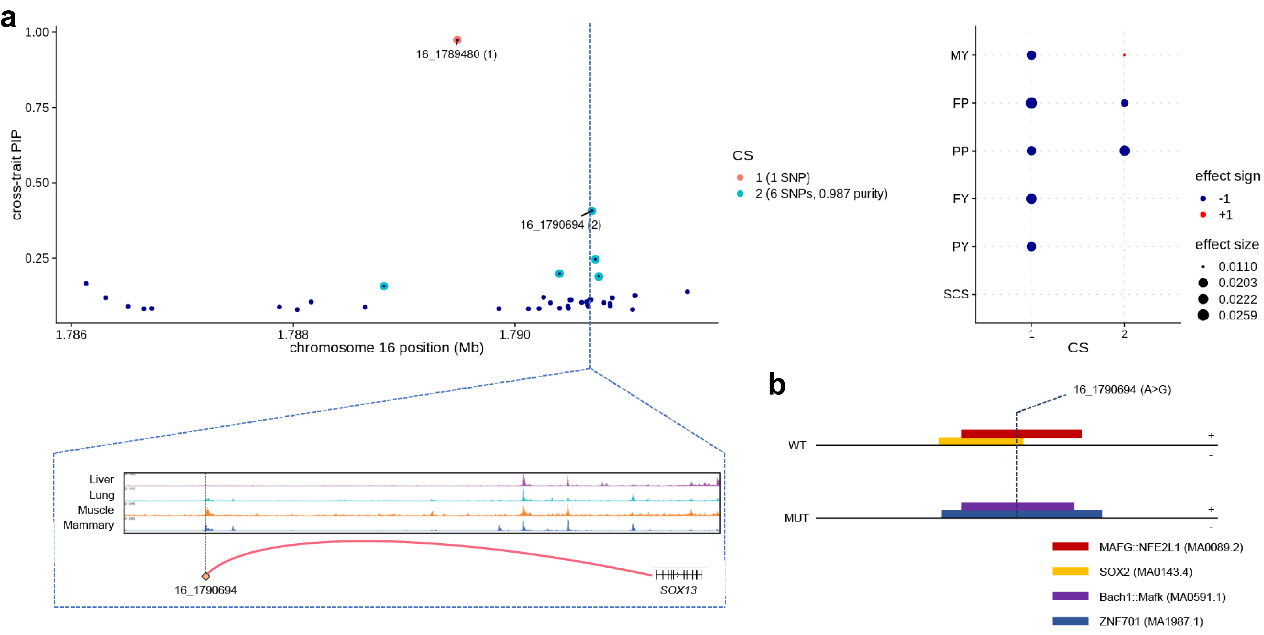


**Figure S7** **Multi-trait Bayesian fine-mapping results and transcription factor binding changes associated with the lead SNP 16_1790694 in the QTL region Chr16:1,786,132-1,791,556.** (**a**) Fine-mapping results for the QTL region Chr16:1,786,132-1,791,556. The lead SNP 16_1790694 which is coincided with ATAC-seq peaks observed in liver, lung, and mammary tissues. MY: milk yield. FP: fat percentage. PP: protein percentage. FY: fat yield. PY: protein yield. SCS: somatic cell score. (**b**) Predicted transcription factor (TF) binding changes caused by SNP 16_1790694 (A>G). The reference allele (WT) and alternative allele (MUT) sequences are shown with predicted TF binding regions.


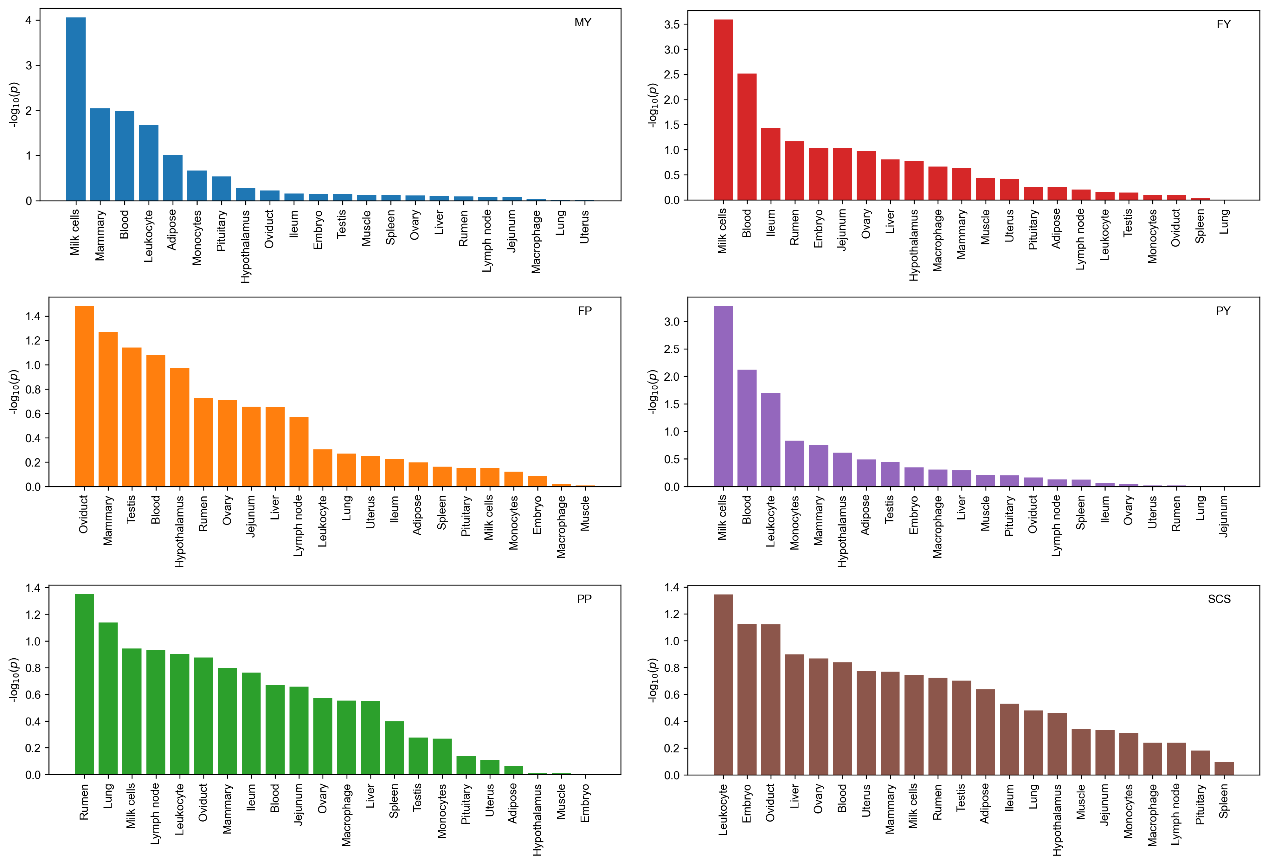


**Figure S8** **The tissue enrichment analysis using MAGMA.** MY: milk yield. FP: fat percentage. PP: protein percentage. FY: fat yield. PY: protein yield. SCS: somatic cell score.


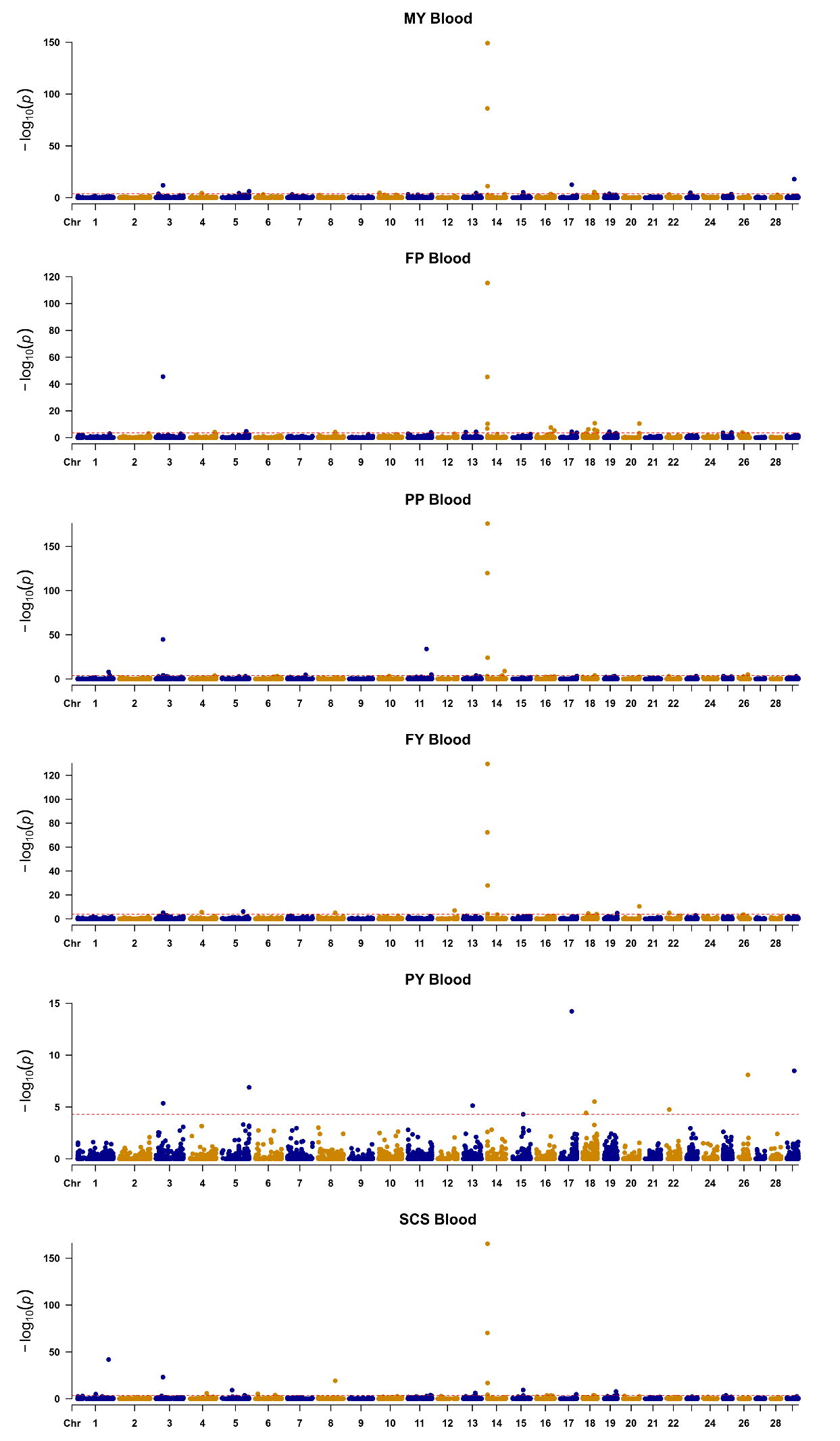


**Figure S9** **Manhattan plots of PMR-Egger results in blood.** The red straight line indicates the significance threshold of the false discovery rate = 0.05. MY: milk yield. FP: fat percentage. PP: protein percentage. FY: fat yield. PY: protein yield. SCS: somatic cell score.


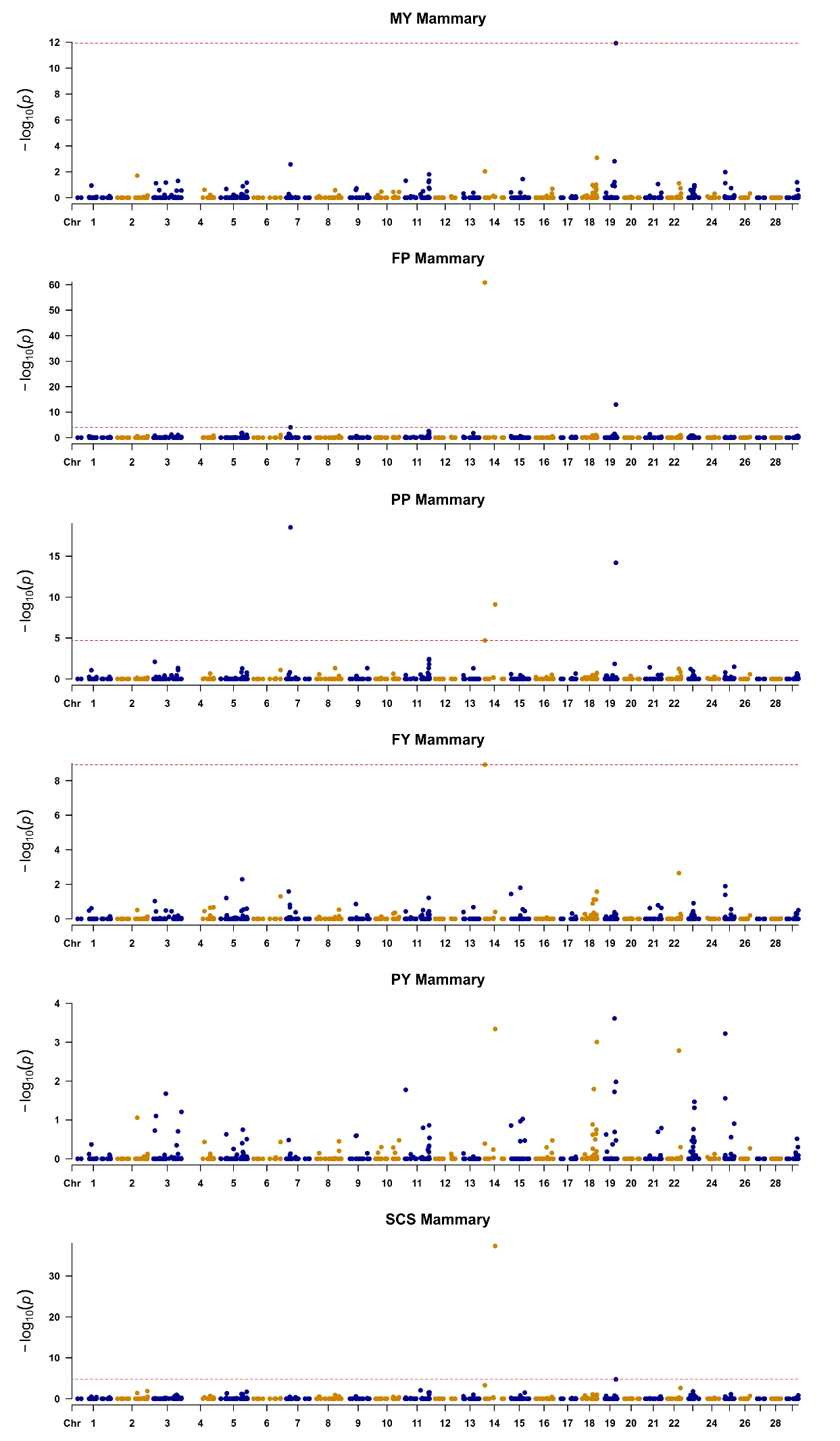


**Figure S10** **Manhattan plots of PMR-Egger results in mammary.** The red straight line indicates the significance threshold of the false discovery rate = 0.05. MY: milk yield. FP: fat percentage. PP: protein percentage. FY: fat yield. PY: protein yield. SCS: somatic cell score.


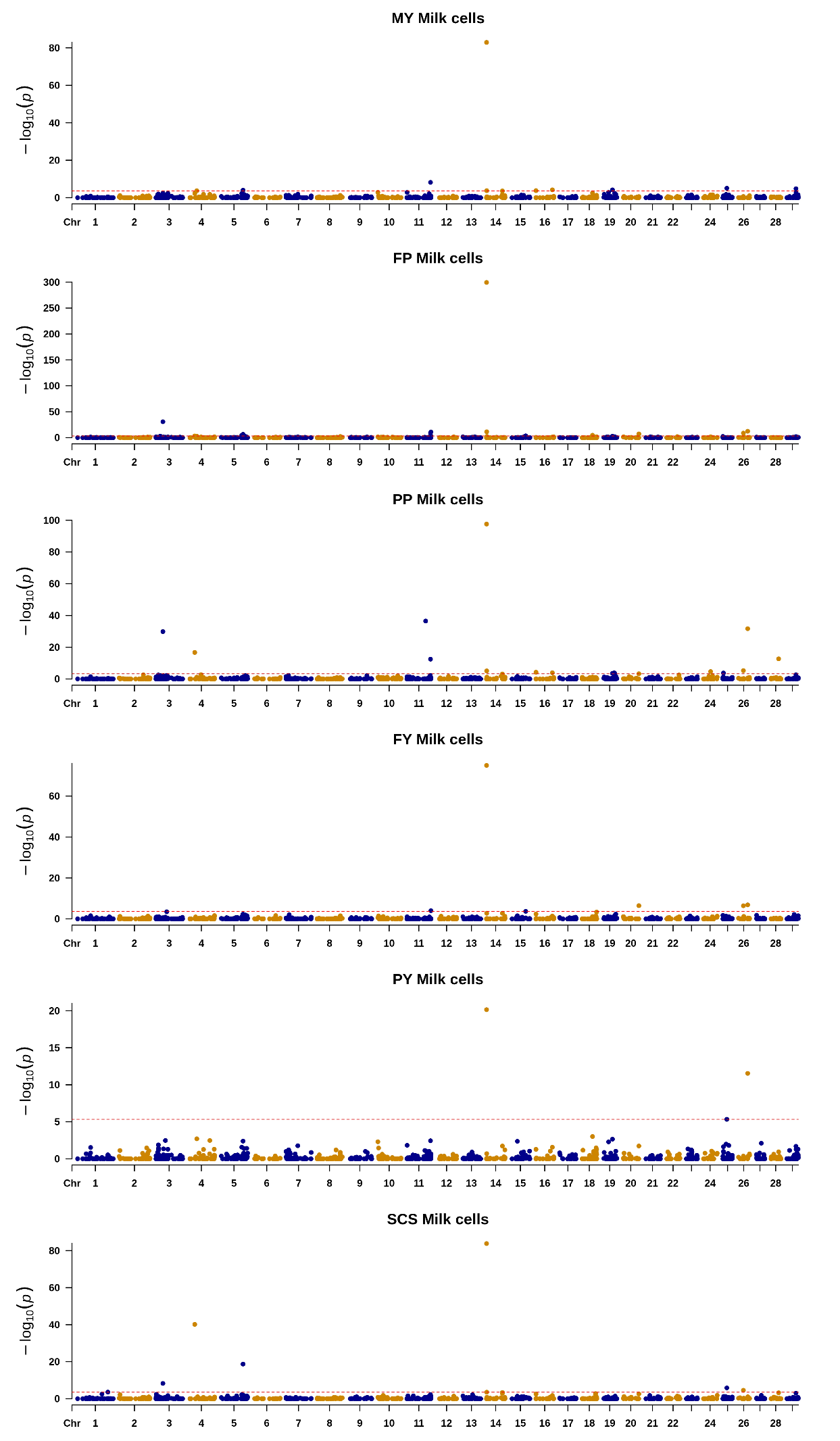


**Figure S11** **Manhattan plots of PMR-Egger results in milk cells.** The red straight line indicates the significance threshold of the false discovery rate = 0.05. MY: milk yield. FP: fat percentage. PP: protein percentage. FY: fat yield. PY: protein yield. SCS: somatic cell score.


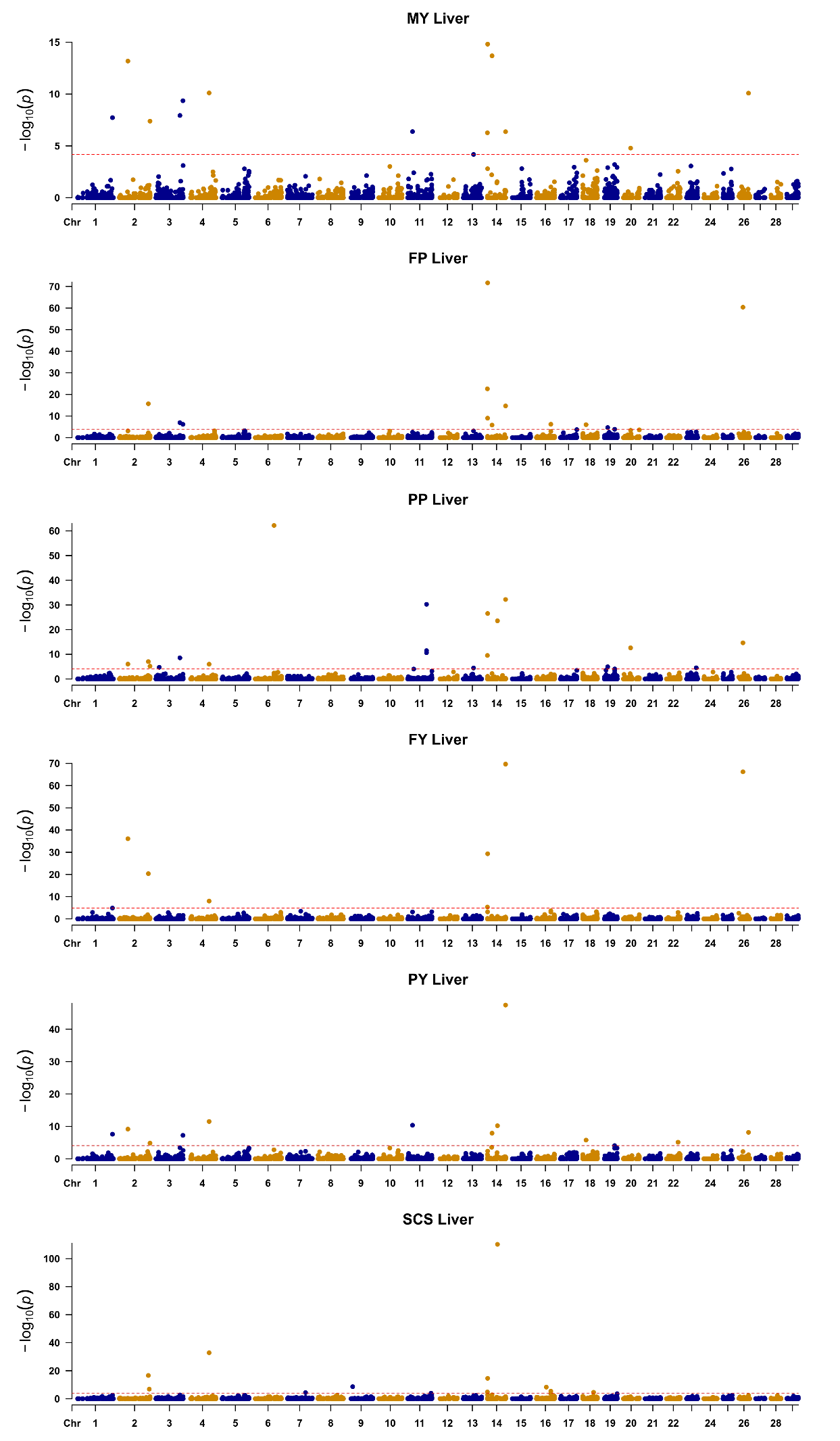


**Figure S12** **Manhattan plots of PMR-Egger results in liver.** The red straight line indicates the significance threshold of the false discovery rate = 0.05. MY: milk yield. FP: fat percentage. PP: protein percentage. FY: fat yield., PY: protein yield. SCS: somatic cell score.


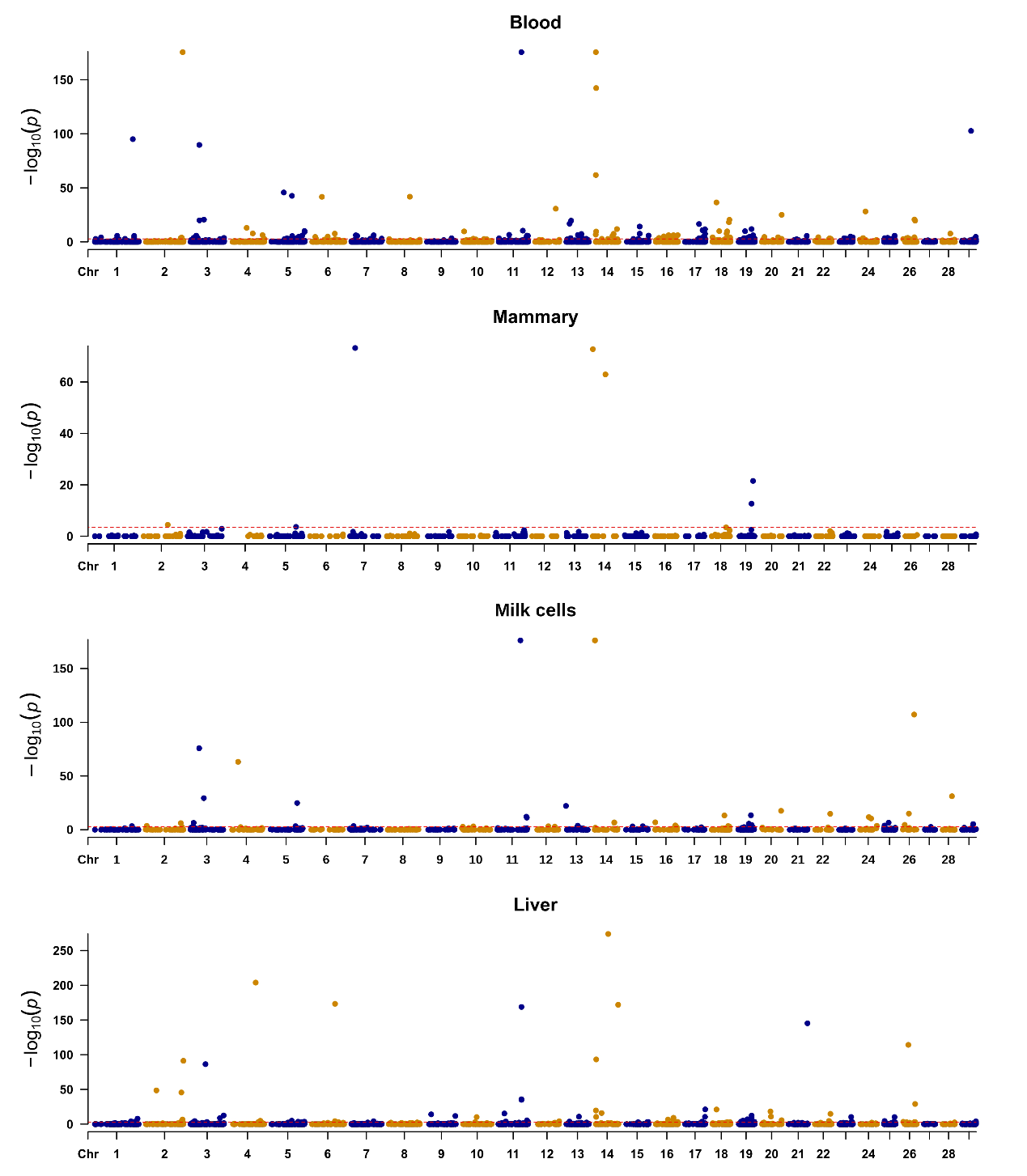


**Figure S1****3 Manhattan plots of moPMR-Egger results in blood, mammary, milk cells, and liver.** The red straight line indicates the significance threshold of the false discovery rate = 0.05.
